# Integrated Microbiome and Metabolomic Profiling to Identify Potential Biomarkers of Major Depressive Disorder

**DOI:** 10.4014/jmb.2512.12014

**Published:** 2026-01-22

**Authors:** Hyunjung Lee, Mee-Hyun Lee, Seung-Ho Seo, Juhan Pak, Soobin Bae, Gayoun Lee, Hyun Sik Kim, Kyeongok Kim, Jae-Hong Kim, Hong-Seok Son

**Affiliations:** 1 Department of Biotechnology, College of Life Sciences and Biotechnology, Korea University, Seoul 02841, Republic of Korea; 2 College of Korean Medicine, Dongshin University, Naju 58245, Republic of Korea; 3 Korean Medicine Convergence Research Institute, College of Korean Medicine, Dongshin University, Naju 58245, Republic of Korea; 4 Department of Biology, College of Science, Kyung Hee University, Seoul 02447, Republic of Korea; 5 Department of Neuropsychiatry, College of Korean Medicine, Dongshin University, Naju 58245, Republic of Korea; 6 Department of Acupuncture and Moxibustion Medicine, College of Korean Medicine, Dongshin University, Naju 58245, Republic of Korea

**Keywords:** Major depressive disorder, Gut microbiota, Metabolomics, Multi-omics, Gut-brain axis

## Abstract

The pathophysiology of major depressive disorder (MDD) remains incompletely understood, hindering the development of objective diagnostic markers. While the microbiota-gut-brain axis is implicated in MDD, the functional link between gut dysbiosis and systemic metabolism remains largely obscure. To address this, we employed an integrated multi-omics approach combining 16S rRNA gene sequencing, GC-MS analysis of urine and plasma, complemented by UPLC-QTOF-MS profiling of plasma, in a Korean cohort (*n* = 69). We identified distinct taxonomic shifts, specifically the enrichment of the *Eubacterium eligens* group and *Veillonella* in MDD patients. Integrated correlation analysis revealed a functional “gut-lipid axis”, where these taxa were strongly associated with alterations in host acylcarnitine and fatty acid metabolism. Notably, diagnostic evaluation demonstrated that the plasma metabolic profile yielded superior predictive accuracy (AUC = 0.862) compared to the gut microbiota (AUC = 0.654). Our findings suggest that while the gut microbiome provides mechanistic insights into lipid dysregulation, the circulating metabolome serves as a more robust, proximal diagnostic readout for MDD.

## Introduction

Major Depressive Disorder (MDD), often referred to as clinical depression, is a debilitating psychiatric condition defined not only by persistent low mood and anhedonia [1], but also by significant disruptions in physical and cognitive health, including sleep, appetite, and concentration [2]. Beyond individual suffering, MDD represents a profound global health challenge, affecting approximately 280 million people—3.8% of the total population and 5.0% of adults [3]—and ranking as a leading cause of disability-adjusted life years (DALYs) and non-fatal health loss worldwide [4]. Notably, reported prevalence rates exhibit substantial regional variability, ranging from 8.3% in the United States [5] to 12.5% in France [6], with similarly distinct patterns observed in Asian populations [7]. This variability may reflect differences in environmental risk factors or inconsistencies in diagnostic reporting, further highlighting the need for objective biological markers [8]. Despite this prevalence and complexity, clinical management remains suboptimal. Currently, the diagnosis of MDD relies primarily on subjective, symptom-based assessments, such as the Diagnostic and Statistical Manual of Mental Disorders, 5th Edition (DSM-5) criteria and clinician-administered scales like the Hamilton Depression Rating Scale (HAM-D) [9]. While standard, these methods are susceptible to clinician bias and patient recall inaccuracy. In contrast, gut microbiota and metabolic signatures offer objective biological markers that can facilitate early detection, enable the differentiation of MDD subtypes, and help elucidate underlying pathophysiology in ways that symptom-based scales cannot. Moreover, current antidepressants yield unsatisfactory remission rates, with one-third of patients unresponsive to first-line therapy [10]. Consequently, identifying robust, objective biomarkers is critical to understanding MDD pathophysiology and developing precise diagnostic tools.

Although the pathophysiology of MDD remains incompletely understood, recent studies have implicated the microbiota-gut-brain axis as a critical regulator of neurobehavioral health [11]. While research has consistently reported gut microbiome dysbiosis in MDD patients [12, 13], the functional mechanisms connecting these microbial shifts to depressive phenotypes remains unclear. Current taxonomic profiling focuses primarily on compositional differences, often failing to elucidate the causal or downstream biological consequences of this dysbiosis.

Meanwhile, metabolomics studies have identified specific metabolic signatures associated with MDD [14]. Studies analyzing urine or plasma have revealed distinct disruptions in amino acid and lipid profiles [15], offering a direct representation of the host's physiological state. These metabolic readouts provide valuable insights into the active systemic biochemical alterations accompanying the disorder. However, these biological domains are often examined in isolation, which represents a major limitation of existing research. There is a paucity of studies that simultaneously integrate taxonomic data with multi-matrix metabolomics, leaving the direct association between gut dysbiosis and systemic metabolic alterations largely obscured.

To extend the current body of knowledge, we employed an integrated systems biology approach that bridges the gut microbiome with systemic metabolism reflected in urine and plasma. Addressing the limited biological coverage of prior single-platform studies, we utilized a multi-platform strategy integrating 16S rRNA gene sequencing with dual-platform metabolomics. This approach combines the resolution of taxonomic profiling with broad chemical coverage-capturing both water-soluble primary metabolites and complex lipids—to offer a comprehensive characterization of the microbiome-metabolome interface.

Leveraging this integrated multi-omics platform, we systematically compared microbial community structure and urine and plasma metabolite profiles of healthy controls (HC) and MDD patients to reconstruct the host-microbe metabolic crosstalk. Ultimately, this study aims to advance our understanding of MDD pathophysiology by elucidating the functional links between gut dysbiosis and systemic metabolic alterations, thereby identifying robust, objective biomarkers that facilitate improved diagnostic accuracy and personalized therapeutic strategies.

## Materials and Methods

### Study Cohort

This study was approved by the Institutional Review Board (IRB) of Dongshin University Korean Medicine Hospital (DSGOH-2024-001) in June 2024. A total of 69 participants, aged 20 to 70 years, were enrolled in this study, comprising 42 healthy controls (HC) and 27 individuals with MDD. Participants were recruited via online and offline advertisements, including posters and banners. All participants provided written informed consent after receiving detailed verbal and written information regarding the study. Symptom severity was assessed using the Korean version of the Beck Depression Inventory-II (K-BDI-II), as shown in Table S1. Following a confirmatory Structured Clinical Interview for DSM-5 (SCID; Table S2) conducted by a board-certified neuropsychiatrist and a psychiatry resident, participants were stratified into two groups: the HC group (K-BDI-II score ≤ 13) and the MDD group (K-BDI-II score ≥ 14). Exclusion criteria included severe medical or psychiatric comorbidities, substance abuse, pregnancy, inflammatory bowel disease, and use of antibiotics, anti-inflammatory drugs, or probiotics within the last 4 weeks. Participant characteristics are presented in Table S3. The study design and reporting followed the Strengthening the Reporting of Observational Studies in Epidemiology (STROBE) guidelines for case-control studies [16]. To ensure data integrity, the order of sample analysis was randomized to minimize batch effects, and investigators were blinded to the group allocation during biological sample processing and data acquisition

### Sample Collection

Fecal, urine, and plasma samples were collected from all participants. Fecal samples were self-collected at home using a stool collection tube with preservation buffer (Noble Bio, Republic of Korea). Urine samples were collected at the hospital from each participant using sterile 50 ml conical tubes. Blood samples were collected into EDTA BD Vacutainer tubes (Becton Dickinson, USA) at the hospital. All specimens were transported to the laboratory in mini cooler bags containing ice packs and subsequently stored at -80°C until analysis.

### 16S rRNA Gene Sequencing and Data Processing

DNA was extracted from fecal samples using the AccuFAST automated system (AccuGene Inc., Republic of Korea) following the manufacturer’s protocol. The V3–V4 hypervariable region of the bacterial 16S rRNA gene was amplified using primers 341F and 806R containing unique dual (UD) index adapter sequences. Amplification was performed using 25 cycles of polymerase chain reaction (PCR) with KAPA HiFi HotStart ReadyMix (Roche Sequencing Solutions, USA). The resulting amplicons (approximately 460 bp) were purified using HiAccuBeads (AccuGene Inc.) for next-generation sequencing (NGS) library preparation. The finalized libraries were pooled at equimolar concentrations and sequenced on an Illumina MiSeq platform using the MiSeq Reagent Kit v3 (600 cycles) (Illumina, USA).

Sequencing reads were processed using DADA2 v1.16 to correct amplification errors, remove noise, and infer exact Amplicon Sequence Variants (ASVs) [17]. Taxonomic classification was assigned using the QIIME 2 framework [18], via a Naïve Bayes classifier trained on the Silva database version 138.1 [19]. To account for variations in library size, sequencing depth was rarefied prior to α- and β-diversity analyses, and taxonomic relative abundance tables were generated from normalized counts.

### Statistical Analysis and Microbiota Identification

Statistical analyses and graphical visualizations were performed in R version 4.4.2. Diversity measures were calculated using the vegan [20] and ape [21] packages. Principal coordinate analysis (PCoA) was conducted using the vegdist and cmdscale functions, whereas permutational multivariate analysis of variance (PERMANOVA) was performed with the adonis2 function, all from the vegan package [20]. Both analyses were carried out using Bray–Curtis and Jaccard distance matrices. Linear discriminant analysis effect size (LEfSe) was employed to identify taxonomic features differentiating HC and MDD groups. To minimize false positives and account for the compositional structure of microbiome data, we also employed analysis of compositions of microbiomes with bias correction (ANCOM-BC) for differential abundance analysis across all taxonomic levels. Taxa with a logarithmic linear discriminant analysis (LDA) score > 2.5 were considered significant. Visualizations were generated using the ggplot2 package [22]. Further statistical evaluations of the gut microbiome were carried out with GraphPad Prism software (version 9.4.1, GraphPad Software Inc., USA). The normality of data distribution was verified using the Shapiro–Wilk test. Welch’s *t*-test was used for normally distributed variables, whereas the Mann-Whitney U test was employed for non-normally distributed data. To control for the false discovery rate (FDR) in high-dimensional comparisons, raw *p*-values for microbial taxa were adjusted using the Benjamini–Hochberg procedure. Statistical significance was defined as an FDR-adjusted *q*-value < 0.05.

### GC-MS Analysis and Data Processing

Untargeted metabolic profiling of urine and plasma was conducted via gas chromatography-mass spectrometry (GC-MS, Shimadzu, Japan), following previously established protocols with minor modifications [23]. Frozen samples were thawed in an ice bath prior to extraction. Urine (100 μl) was extracted with 900 μl of methanol, while plasma (40 μl) was extracted with 250 μl of 70% methanol followed by a 30 min incubation at 37°C. After centrifugation at 4°C (15,928 ×*g* for 10 min for urine; 13,570 ×*g* for 5 min for plasma), the resulting supernatants (400 μl for urine, 210 μl for plasma) were collected and mixed with 20 μl of ribitol internal standard (0.5 mg/ml). A pooled quality control (QC) sample was prepared using 30 μl from each extract, and all samples were dried using a vacuum concentrator (HyperVAC, Republic of Korea) for 12 h.

After vacuum drying, the samples were derivatized with 100 μl of O-methoxyamine hydrochloride (20 mg/ml in pyridine) and ultrasonicated using a Powersonic 520 (Hwashin, Republic of Korea) at 4°C for 20 min to ensure complete dissolution. Each sample was vortexed for 1.5 min and incubated in the dark at 30°C while shaking at 75 rpm for 90 min. After incubation, 50 μl of N-methyl-N-trimethylsilyl-trifluoroacetamide (MSTFA) was added. The samples were briefly vortexed and subjected to another incubation in the dark at 37°C while shaking at 75 rpm for 30 min. Subsequently, the samples were centrifuged at 13,572 ×*g* for 5 min at 4°C, and 80 μl of the supernatant was transferred into a 2 ml glass vial equipped with a 100 μl insert for GC-MS analysis.

The derivatized samples were analyzed using an RTX-5MS capillary column (Restek, USA) coupled to a GC-MS (QP2020, Shimadzu, Japan). The instrument conditions were set as follows: injector temperature of 230°C, transfer line temperature of 250°C, and detector temperature of 280°C. Helium was used as the carrier gas at a flow rate of 1 ml/min. The oven temperature was programmed at 80°C (held for 2 min), increased at a rate of 15°C/min to a final temperature of 330°C (held for 6 min). To ensure stability and reproducibility, QC and blank samples (BS) were analyzed at the beginning of the batch prior to study samples and injected after every 20 samples during analysis.

Raw chromatographic and mass spectral data were acquired using Shimadzu GC Solution software (Shimadzu), which was then converted to ABF files using a file converter. Data processing—including raw peak extraction, baseline filtering, alignment, deconvolution, and integration—was performed using MS-DIAL (ver. 4.9.221218) and an open-source electron ionization (EI) spectral library [24]. For accurate peak identification, retention indices (RI) were calculated based on the retention times of C7–C40 alkane standards (Sigma-Aldrich, USA) analyzed under identical conditions.

Metabolite identification was based on EI-MS spectra and Kovats RI, applying a tolerance of 20 units and a 90% threshold for both EI similarity and identification scores. Putative identifications were further validated against the NIST v. 20.0 library and authentic standard reagents. Peak intensities were normalized first to the internal standard (ribitol) and subsequently by total peak area (TPA) to account for variations in urine and plasma concentrations. Prior to statistical analysis, data quality was refined by excluding features with an average intensity < 1 in QC or BS, as well as metabolites exhibiting poor reproducibility, defined as a relative standard deviation (RSD) > 30%, across pooled QC samples.

### UPLC-QTOF-MS Analysis and Data Processing

Untargeted metabolic profiling of plasma was conducted utilizing ultra-performance liquid chromatography-quadrupole time-of-flight mass spectrometry (UPLC-QTOF-MS, Waters Co., USA). UPLC-QTOF-MS conditions and analysis protocol workflow were adapted from a prior study with minor modifications [25]. Frozen samples were thawed in an ice bath prior to extraction. Metabolites were extracted by mixing 60 μl of plasma with 180 μl of acetonitrile:methanol (4:1, v/v) solvent, followed by 5 min of vortexing and 20 min of ultrasonication at 4°C using Powersonic 520 (Hwashin). The mixture was then centrifuged at 15,928 ×*g* for 10 min at 4°C to isolate the supernatant. To ensure the complete removal of particulates, this centrifugation step was repeated twice before final collection. Subsequently, 150 μl of the supernatant was collected for UPLC-QTOF-MS analysis.

Analysis of plasma extracts was conducted using an ACQUITY UPLC system (Waters, USA) coupled with a Xevo G3 Q-TOF mass spectrometer (Waters) via an electrospray ionization (ESI) source. Chromatographic separation was achieved using an ACQUITY UPLC HSS T3 column (2.1 × 100 mm, 1.8 μm). The mobile phases comprised (A) water and (B) acetonitrile, both containing 0.1% formic acid. The elution gradient was programmed as follows: 0–6 min, 5% B; 6–9 min, 5–95% B; 9–15 min, 95% B; 15–17.1 min, 95–5% B; and 17.1–20 min, 5% B. The flow rate was set to 0.3 ml/min with an injection volume of 2 μl. Mass spectrometry was performed in both positive and negative ionization modes under the following conditions: source temperature, 100°C; desolvation temperature, 300°C; desolvation gas flow, 600 l/h; cone gas flow, 50 l/h; and capillary voltage, 2.5 kV.

Data were acquired over a mass range of m/z 50–1,200 with a scan time of 0.2 sec. Leucine enkephalin (m/z 556.2771 [M+H]+ and 554.2615 [M−H]−) was used as the lockmass for real-time calibration. The collision energy was set to 6 V for the low-energy function and ramped from 25 to 50 V for the high-energy function. All acquisitions were performed in resolution mode with the dynamic range set to normal. To monitor system stability and reproducibility, QC samples were prepared by pooling equal aliquots of each sample and analyzed at regular intervals.

Raw data from UPLC-QTOF-MS data were processed using Progenesis QI (Nonlinear Dynamics, UK). To ensure analytical robustness, features were subjected to a rigorous QC filtering process. Background signals predominantly detected in BS were removed, and feature linearity was verified using a QC dilution series. Additionally, precision filtering was applied based on the RSD of the pooled QC samples.

Metabolite annotation was conducted by matching accurate mass, isotopic distributions, and fragmentation spectra against public databases. Only candidates exceeding a defined annotation score threshold were retained. Final identifications were confirmed based on mass accuracy, fragmentation pattern agreement, and isotope similarity. Subsequently, structural identification and biological interpretation were conducted via exact mass searches in metabolite databases, including the Human Metabolome Database (HMDB; https://www.hmdb.ca/, accessed on 10 Nov 2025) and PubChem (https://pubchem.ncbi.nlm.nih.gov/, accessed on 10 Nov 2025). System reproducibility was monitored via pooled QC samples.

### Statistical Analysis and Metabolite Identification

Multivariate statistical analysis, specifically partial least squares discriminant analysis (PLS-DA), was conducted using SIMCA v.18.0 (Umetrics, Sweden). The validity of the PLS-DA model was evaluated using *R*^2^ and *Q*^2^ values, and model robustness was confirmed via permutation testing (*n* = 999). To identify metabolites significantly contributing to group classification, features with a variable importance in projection (VIP) score > 1.0 were selected from the PLS-DA loading plot. Further statistical evaluations of the urine and plasma metabolites were conducted using GraphPad Prism software (version 9.4.1, GraphPad Software Inc., USA). The normality of the data distribution was assessed using the Shapiro–Wilk test. Normally distributed variables were compared using Welch’s *t*-test, while non-normally distributed variables were analyzed using the Mann-Whitney U test. To address multiple comparisons in high-dimensional data, raw *p*-values for metabolites were adjusted using the FDR method. Significance was established at an FDR-corrected *q*-value < 0.05

### Integrative Analysis and Biomarker Evaluation

Metabolite set enrichment analysis (MSEA) was conducted via quantitative enrichment analysis using MetaboAnalyst 6.0 (https://www.metaboanalyst.ca, accessed on 12 Nov. 2025). Analysis of the urinary metabolome was based exclusively on processed GC-MS data. For plasma, processed GC-MS and UPLC-QTOF-MS datasets (positive and negative modes) were normalized to z-scores and merged prior to analysis to ensure data compatibility.

Spearman’s rank correlation analysis was performed to evaluate relationships between gut microbiota (genus level) and metabolic biomarkers in plasma derived from UPLC-QTOF-MS. The resulting correlations were visualized as heatmaps, where color intensity represented the correlation coefficient (ρ). Statistical significance was indicated as follows: *p < 0.05, **p < 0.01, and ***p < 0.001.

Diagnostic accuracy was evaluated using Receiver Operating Characteristic (ROC) analysis via the pROC package [26]. For the combined model, features were selected based on significant associations identified in the Spearman’s rank correlation analysis. To mitigate overfitting, we employed 5-fold cross-validation with ridge logistic regression (α = 0). Model performance was quantified using the Area Under the Curve (AUC) with 95% confidence intervals, and all visualizations were generated using ggplot2 [22].

## Results

### Taxonomic Composition and Diversity Assessment of Gut Microbiota

**Fig. 1:**
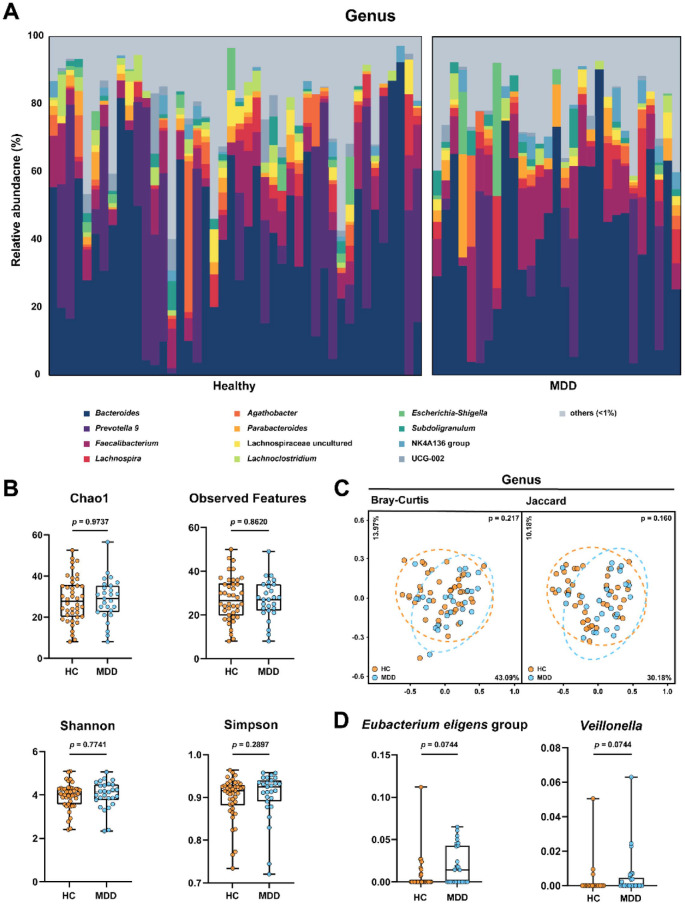
Gut microbiota composition and diversity analysis in HC and MDD groups. (**A**) Relative abundance of bacterial taxa at the genus level for each individual in HC and MDD groups. (**B**) Comparison of alpha diversity indices (Chao1, Observed Features, Shannon, and Simpson) between HC and MDD cohorts. (**C**) Principal coordinate analysis (PCoA) score plots based on Bray-Curtis dissimilarity and Jaccard distance matrices. Statistical significance was assessed using PERMANOVA. (**D**) Box plots illustrating the relative abundance of the genus level taxa with marginally significant difference (*q* < 0.10) between healthy and MDD groups.

We investigated the taxonomic composition of gut microbiota to determine the potential difference between HC and MDD. A total of 42 healthy controls and 27 MDD patients were included in the final analysis, with no exclusions. Substantial inter-individual variability in microbial composition was observed at the genus level in Fig. 1A, but there was no discernible clustering or pattern between the two groups. Both groups shared the same four most dominant genera—*Bacteroides*, *Prevotella* 9, *Faecalibacterium*, and *Lachnospira*—but the order of their abundance differed. While *Prevotella* 9 (a specific genus-level group within the Prevotellaceae family defined by the SILVA database) was the second most abundant genus in HC, it was surpassed by *Faecalibacterium* in the MDD group.

To further investigate the structure of microbiota, we analyzed the α-diversity, which reflects the species richness and evenness of individuals, using Chao1, Observed Features, Shannon, and Simpson indices (Fig. 1B). No significant difference between the two-cohort groups was observed, suggesting that the overall microbial diversity of the two groups is similar.

In order to characterize differences in microbial community structure between groups, we calculated β-diversity using both the Bray-Curtis and Jaccard indices, which were then presented through PCoA (Fig. 1C). At the genus level, HC and MDD groups exhibited similar community compositions with substantial overlap and no significant clustering (PERMANOVA, *p* > 0.05 for all comparisons). This result suggests there were no clear differences between the HC and MDD groups at the community level.

Therefore, univariate statistical testing was applied at genus-level taxa, comparing the abundance of each genus within the two groups. Although two genus-level taxa, *E. eligens* group and *Veillonella*, showed strong nominal significance (*p* = 0.0029 and *p* = 0.0031, respectively), they did not remain statistically significant after FDR correction (*q* = 0.0744) (Fig. 1D). However, they displayed a notable trend with the MDD group harboring a relatively higher abundance of these two genera. Corresponding raw *p*-values, FDR-adjusted *q*-values, and effect sizes (Hedges’ *g*) of genus-level taxa are provided in Table S4.

### Differential Taxonomic Features of Gut Microbiota

**Fig. 2:**
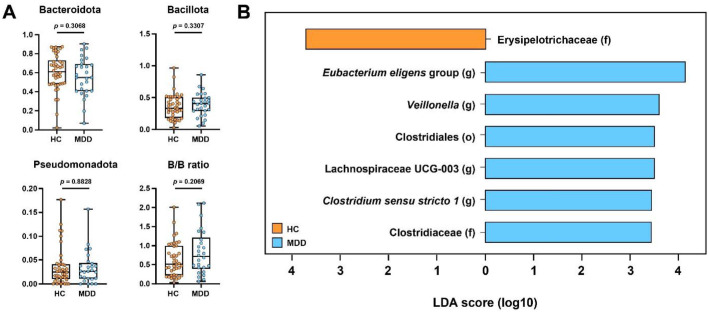
Phylum-level comparison and identification of discriminatory taxa using LEfSe analysis. (**A**) Comparison of the relative abundance of major bacterial phyla between HC and MDD groups. The B/B ratio represents the ratio of Bacillota to *Bacteroidota* relative abundance. (**B**) Linear Discriminant Analysis Effect Size (LEfSe) histogram distinguishing the gut microbiota of MDD patients from healthy controls.

We compared the relative abundance of bacterial phyla between HC and MDD patients (Fig. 2A). However, no significant differences were observed between the two groups. Furthermore, the Bacillota (formerly *Firmicutes*) to *Bacteroidota* ratio, a common marker of gut dysbiosis, did not differ significantly between HC and MDD patients. This suggests that MDD-associated dysbiosis is not characterized by a broad restructuring of dominant phyla, indicating that differences—if present—may occur at finer taxonomic levels rather than at the phylum scale.

To further investigate these finer alterations and identify specific discriminatory taxa distinguishing the HC from the MDD group, we performed LEfSe analysis on the 16S rRNA amplicon sequencing data at taxonomic levels from phylum to genus, applying LDA score threshold of 2.5 (Fig. 2B). Four bacterial genera (*E. eligens* group, *Veillonella*, Lachnospiraceae UCG-003, and *Clostridium* sensu stricto 1), one bacterial family (Clostridiaceae), and one bacterial order (Clostridiales) were enriched in the MDD group. Conversely, the family Erysipelotrichaceae was significantly enriched in the HC group.

### GC-MS Based Urinary Metabolic Profiles and Differential Metabolites Identification

**Fig. 3:**
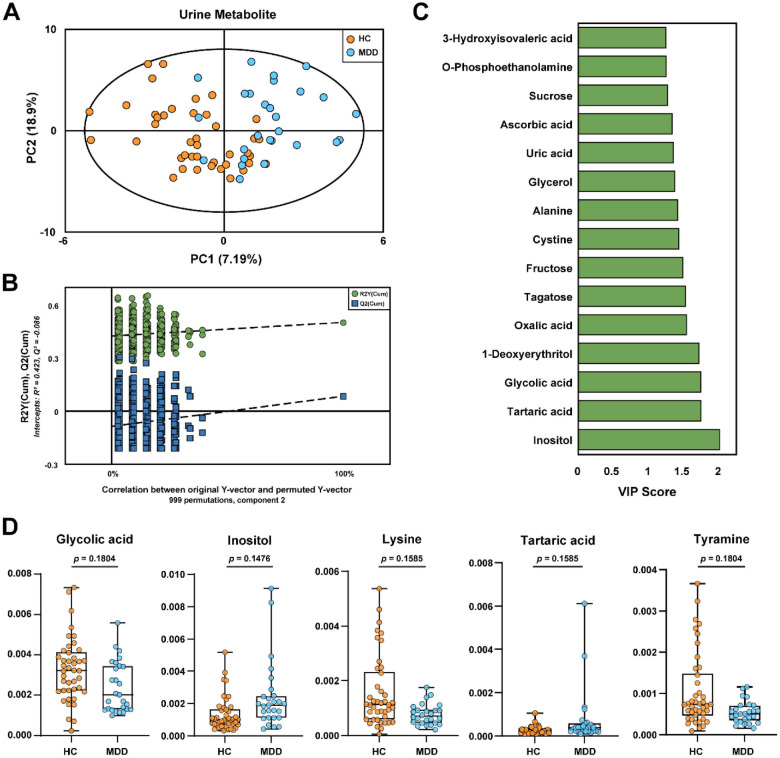
GC-MS based urinary metabolomics analysis distinguishing MDD patients from healthy controls. (**A**) Partial least squares discriminant analysis (PLS-DA) score plot showing the separation between HC and MDD groups. (**B**) Validation of the PLS-DA model using permutation testing (n = 999). (**C**) VIP scores (> 1.0) of the top 15 metabolites contributing to the discrimination between groups. (**D**) Box plots illustrating the relative abundance of representative differential metabolites (*q* < 0.20) between HC and MDD groups.

We conducted GC-MS analysis of urinary metabolites to determine metabolic profiles that differentiate the MDD group from the HC group. The PLS-DA plot revealed a clear separation between HC and MDD groups (Fig. 3A). Model reliability was further verified by permutation analysis (*n* = 999), in which the permuted *R*^2^ and *Q*^2^ distributions were significantly reduced relative to the original values, and the negative *Q*^2^ intercept (− 0.086) indicated that the PLS-DA model was not overfitted (Fig. 3B). A total of 32 metabolites were identified as significant contributors to group differentiation (VIP > 1.0; Table S5). The top 15 discriminatory metabolites based on VIP scores are visualized in Fig. 3C. Among the 32 metabolites with VIP > 1.0, we identified five metabolites–glycolic acid, inositol, lysine, tartaric acid, and tyramine–with strong nominal significance (*p* < 0.05). Although these differences did not remain significant after a strict FDR threshold of 0.05, the specific trends are notable (Fig. 3D). Levels of glycolic acid, lysine, and tyramine were lower in MDD patients, while levels of inositol and tartaric acid were higher compared to healthy controls. A comprehensive summary of univariate statistics, including raw *p*-values, FDR-corrected *q*-values, and Hedges’ *g* effect sizes, is presented in Table S6.

### GC-MS Based Plasma Metabolic Profiles and Differential Metabolites Identification

**Fig. 4:**
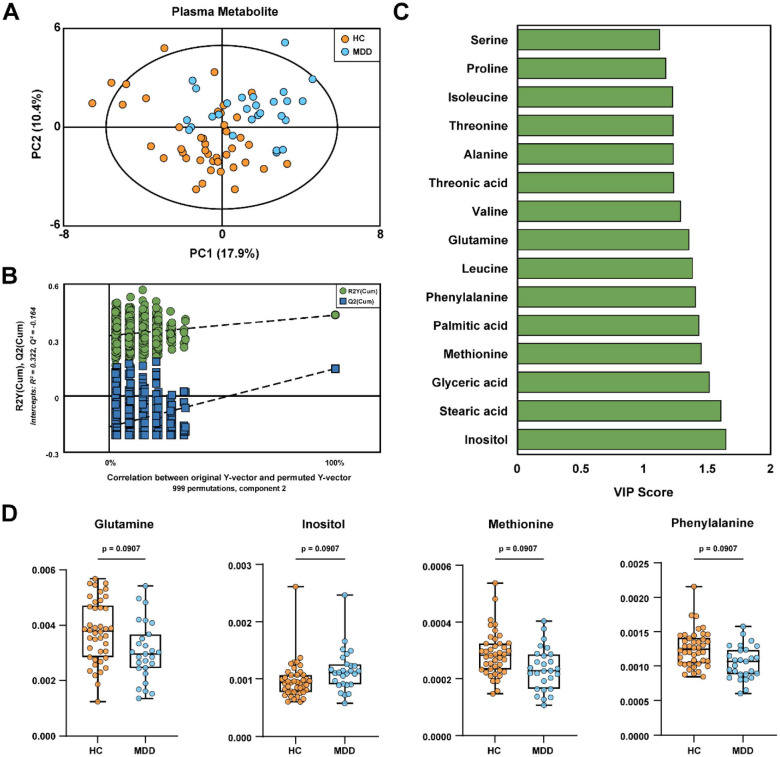
GC-MS based plasma metabolomics analysis distinguishing MDD patients from healthy controls. (**A**) Partial least squares discriminant analysis (PLS-DA) score plot showing the separation between HC and MDD groups. (**B**) Validation of the PLS-DA model using permutation testing (n = 999). (**C**) VIP scores (> 1.0) of the top 15 metabolites contributing to the discrimination between groups. (**D**) Box plots illustrating the relative abundance of representative differential metabolites (*q* < 0.10) between HC and MDD groups.

To evaluate systemic metabolic alterations associated with MDD, we performed GC-MS-based metabolomic analysis of plasma. Multivariate modeling using PLS-DA showed a clear distinction between HC and MDD groups (Fig. 4A). The robustness of this model was supported by permutation testing (*n* = 999), where the *Q*^2^ intercept of − 0.164 ruled out overfitting (Fig. 4B). Based on a VIP threshold of > 1.0, we identified 18 metabolites responsible for group separation (Table S5), with the top 15 illustrated in Fig. 4C. Among these, four metabolites—glutamine, inositol, methionine, and phenylalanine—displayed nominal statistical significance (*p* < 0.01) and *q* value lower than 0.10 (Fig. 4D). While these features did not satisfy the strict FDR correction, they exhibited meaningful biological trends; specifically, the amino acids glutamine, methionine, and phenylalanine were reduced in the MDD group relative to controls. Detailed univariate statistics, including raw *p*-values, FDR-adjusted *q*-values, and effect sizes (Hedges’ *g*), are summarized in Table S7.

### UPLC-QTOF-MS Based Plasma Metabolic Profiles and Differential Metabolites Identification

**Fig. 5:**
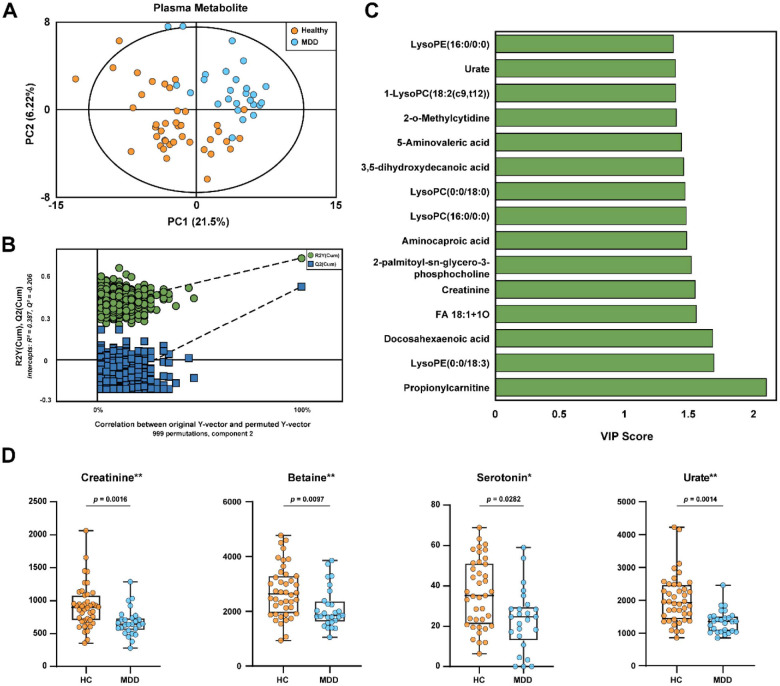
UPLC-QTOF-MS based plasma metabolomics analysis distinguishing MDD patients from healthy controls. (**A**) Partial least squares discriminant analysis (PLS-DA) score plot showing the separation between HC and MDD groups. (**B**) Validation of the PLS-DA model using permutation testing (*n* = 999). (**C**) VIP scores (> 1.0) of the top 15 metabolites contributing to the discrimination between groups. (**D**) Box plots illustrating the relative abundance of representative differential metabolites (*q* < 0.05) between HC and MDD groups.

While GC-MS provides comprehensive coverage of primary water-soluble metabolites (predominant in urine), it has limited sensitivity for larger, non-volatile, and hydrophobic species. Therefore, to specifically expand our coverage of the plasma metabolome to include lipids and secondary metabolites, we employed UPLC-QTOF-MS to complement the GC-MS results. Multivariate analysis using PLS-DA revealed clear clustering that distinguished HC and MDD groups (Fig. 5A). Permutation testing (*n* = 999) supported model validity, as both *R*^2^ and *Q*^2^ values from the permuted datasets were substantially lower than the original model, and the negative *Q*^2^ intercept (− 0.206) confirmed that overfitting was unlikely (Fig. 5B). Applying a VIP cutoff of > 1.0, we identified 46 plasma metabolites contributing most to the separation between MDD and HC subjects (Table S8). The top 15 most discriminatory features are ranked in Fig. 5C. For the identification of differential metabolites, we prioritized features with high statistical confidence and well-established relevance to MDD pathophysiology, independent of their VIP ranking. We identified four key metabolites—creatinine, betaine, serotonin, and urate—that exhibited robust statistical significance (*p* < 0.05) and passed the strict FDR correction (*q* < 0.05) (Fig. 5D). Levels of betaine, creatinine, serotonin, and urate were significantly lower in the MDD group. Full statistical metrics for these features, including calculated effect sizes and adjusted *q*-values, are cataloged in Tables S8 and S9.

### Key Metabolic Pathway Analysis for Plasma and Urinary Metabolites

**Fig. 6:**
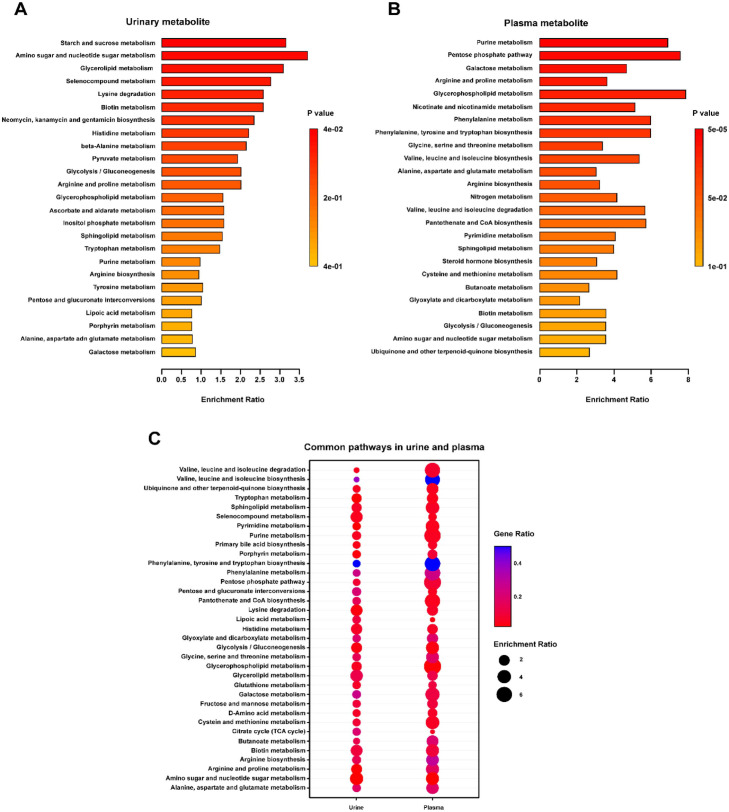
Enrichment analysis using urinary and plasma metabolite profiles. Overview of metabolite set enrichment for (**A**) plasma and (**B**) urine. The enrichment ratio represents the proportion of metabolites detected within each pathway. (**C**) Comparative KEGG pathway analysis of the 35 dysregulated metabolic pathways shared between urine and plasma.

To elucidate the biological functions associated with the identified differential metabolites, we performed metabolite set enrichment analysis (MSEA) on urinary and plasma metabolites. In the urinary metabolome, metabolic alterations were primarily concentrated in carbohydrate and lipid metabolism (Fig. 6A). Specifically, starch and sucrose metabolism and amino sugar and nucleotide sugar metabolism showed the highest enrichment ratios. Detailed analysis revealed that the enrichment in starch and sucrose metabolism was driven by higher levels of sucrose in MDD, suggesting impaired carbohydrate digestion or absorption. Similarly, the amino sugar pathway was characterized by increased concentrations of glucose and galactose, indicating a potential accumulation of simple sugars due to disrupted glycolysis or altered microbial consumption. Lysine degradation and glycerolipid metabolism were also significantly impacted (*p* < 0.05), with key metabolites such as lysine and glycerol showing downregulation relative to controls.

The plasma metabolome revealed widespread metabolic dysregulation with highly significant pathway enrichments (Fig. 6B). The most prominently altered pathways included purine metabolism, the pentose phosphate pathway, and galactose metabolism, all of which exhibited high enrichment ratios and robust statistical significance (*p* < 0.001). Specifically, the disruption in purine metabolism was marked by depleted levels of glutamine, inosine, and uric acid, pointing to reduced antioxidant capacity and altered nitrogen balance in MDD patients. Furthermore, consistent with our findings, glycerophospholipid metabolism was identified as the significantly enriched pathway with the highest enrichment ratio in plasma. This pathway alteration was primarily defined by lower abundance of Lyso PC (18:1/0:0) and elevated inositol levels, reflecting potential disturbances in lipid signaling and cell membrane stability associated with depressive pathology.

To determine whether these metabolic shifts were fluid-specific or systemic, we compared the enrichment profiles of urine and plasma and found 35 common pathways (Fig. 6C). Notably, several pathways-including glycerophospholipid metabolism, alanine, aspartate, and glutamate metabolism, and amino sugar and nucleotide sugar metabolism-were perturbed in both biofluids. Mechanistically, the shared dysregulation in glycerophospholipid metabolism involved consistent elevations in inositol levels across both plasma and urine, reinforcing the systemic nature of the observed lipidome disruption. This overlap suggests that MDD is characterized by a systemic metabolic disruption affecting multiple biological compartments, rather than isolated tissue-specific changes.

### Correlation Analysis between Microbial and Metabolic Profiling

**Fig. 7:**
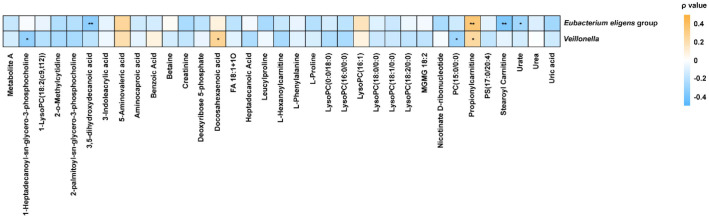
Spearman correlation heatmap illustrating the functional associations between gut microbiota and plasma metabolites. The analysis was performed on the key discriminatory genus-level taxa (*q* < 0.10) and plasma metabolites satisfying statistical criteria of VIP > 1.0 and FDR-corrected *q* < 0.05. Statistical significance is denoted by asterisks: *p < 0.05 and **p < 0.01. Note: 'Metabolite A' represents the compound (2R,3R,4R,6aR,6bS,8aR,14bR)-2,3,12-trihydroxy-4,6a,6b,11,11,14b-hexamethyl-8a-[(2S,3R,4S,5S,6R)-3,4,5-trihydroxy-6-(hydroxymethyl)oxan-2-yl]oxycarbonyl-1,2,3,4a,5,6,7,8,9,10,12,12a,14,14atetradecahydropicene-4-carboxylic acid, , abbreviated for graphical clarity.

To investigate the potential functional interplay between the gut microbiome and host metabolism, we performed a Spearman correlation analysis presented in a heatmap (Fig. 7). For this analysis, we specifically selected features that demonstrated the strongest statistical signals in our previous steps: genus-level taxa with *q* < 0.10 and metabolites satisfying both VIP > 1.0 and *q* < 0.05. The analysis revealed distinct patterns of metabolic association, particularly involving lipid-related molecules and fatty acid derivatives. The *E. eligens* group exhibited a significant positive correlation with propionylcarnitine (*r* = 0.342, *q* = 0.008). In contrast, the taxa displayed strong negative correlations with the long-chain acylcarnitine stearoyl carnitine (*r* = − 0.378, *q* = 0.003). This negative relationship with stearoyl carnitine is particularly notable; given that *Eubacterium eligens* group was enriched in the MDD patients, this inverse correlation aligns with reported reductions of acylcarnitine in depression [27]. Additionally, this genus was negatively associated with the fatty acid derivative 3,5-dihydroxydecanoic acid (*r* = − 0.338, *q* = 0.009) and urate (*r* = − 0.278, *q* = 0.042). Similarly, *Veillonella* showed a positive association with propionylcarnitine (*r* = 0.239, *q* = 0.048). This consistency between both genera suggests a robust link between the gut microbiota and short-chain acylcarnitine pools, potentially driven by bacterial propionate production [28]. *Veillonella* was also positively correlated with docosahexaenoic acid (*r* = 0.270, *q* = 0.049). Conversely, it exhibited negative correlations with specific lipids, including PC (15:0/0:0) (*r* = − 0.289, *q* = 0.032) and 1-heptadecanoyl-sn-glycero-3-phosphocholine (*r* = − 0.312, *q* = 0.018). These findings suggest that the abundance of specific gut bacteria in MDD is tightly linked to alterations in systemic lipid composition and fatty acid transport mechanisms.

### Predictive Performance of Gut Microbiota and Metabolites for MDD Classification

**Fig. 8:**
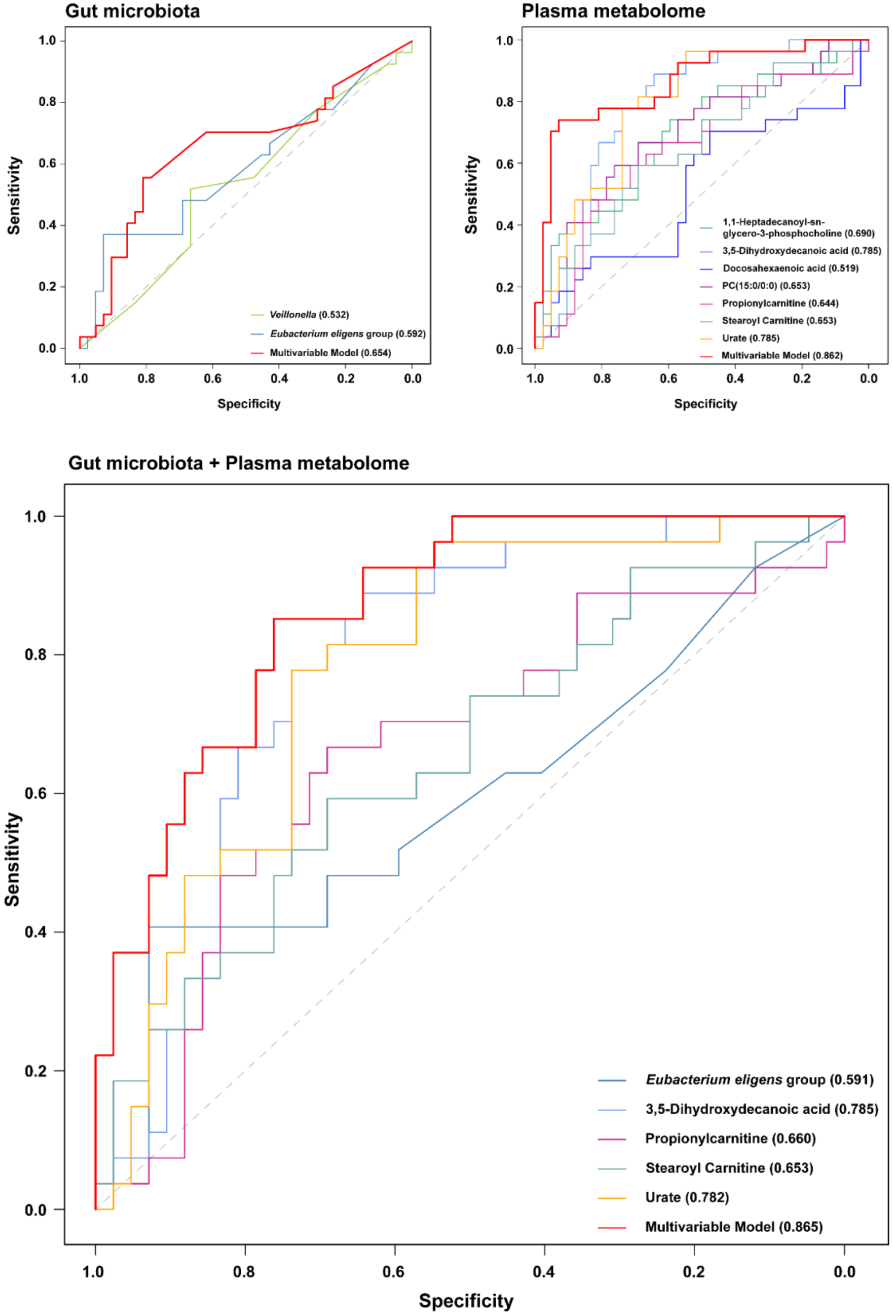
Evaluation of diagnostic performance using receiver operating characteristic (ROC) analysis. ROC curves illustrating the classification accuracy of individual biomarkers and multivariate models based on the (**A**) gut microbiota and (**B**) plasma metabolome. The red lines represent the composite multivariate models for each dataset, while other colored lines represent specific contributing features. (**C**) Performance of the integrated combinatorial model. The graph displays the specific features retained in the final model alongside the resulting multivariate curve (red line). Area under the curve (AUC) values for each variable are listed in the figure key.

To evaluate the clinical utility of the identified candidate biomarkers, we constructed ROC curves for the gut microbiota and plasma metabolites, both separately and in combination. The diagnostic performance of the gut microbiota alone was relatively limited (Fig. 8A). The individual AUC values for *Veillonella* (0.532) and the *E. eligens* group (0.592) suggested weak discriminatory power, and even the multivariate microbial model achieved only a modest AUC of 0.654. In contrast, the plasma metabolic profile demonstrated superior diagnostic accuracy (Fig. 8B). Individual metabolites derived from the UPLC-QTOF-MS analysis, such as 3,5-dihydroxydecanoic acid and urate, achieved impressive AUCs of 0.785, while the multivariate plasma model reached an AUC of 0.862. The stronger performance of the plasma model likely reflects the fact that circulating metabolites represent the functional output of systemic physiology, which is more tightly regulated and proximal to the disease phenotype than the highly variable gut microbiome.

We next assessed whether integrating the gut and plasma datasets could enhance classification accuracy. Based on the functional correlations identified in Fig. 7, we constructed combinatorial models. Adding *Veillonella* to its correlated metabolites did not improve the diagnostic yield. This suggests that the diagnostic information carried by *Veillonella* is largely redundant to the plasma markers; essentially, the systemic metabolic shift (*e.g.*, altered carnitines) already captures the signal provided by the bacteria. However, a specific multivariable model combining the *E. eligens* group with four key metabolites (3,5-dihydroxydecanoic acid, propionylcarnitine, stearoyl carnitine, and urate) yielded the highest overall performance, with an AUC of 0.865 (Fig. 8C). While this represents only a marginal numeric increase (+ 0.003) over the plasma-only model (0.862), it indicates that *E. eligens* group provides complementary diagnostic information. The retention of *E. eligens* group in the final model suggests that this taxon captures a specific aspect of the host-microbiome interaction—likely related to inflammation or gut barrier integrity—that is not fully encapsulated by the plasma lipid profile alone.

## Discussion

In this study, we addressed the need for a system-level understanding of MDD by integrating microbiome and metabolomic datasets to reconstruct the host-microbe metabolic crosstalk. Our findings answer the question of pathophysiology by revealing that MDD is defined not by broad microbial dysbiosis, but by a targeted disruption of the “gut-lipid axis”, where specific taxonomic shifts are significantly associated with alterations in host energy metabolism. Furthermore, regarding the search for objective diagnostic indicators, we demonstrated that the gut microbiome and systemic metabolome do not contribute equally to diagnostic accuracy; rather, the plasma metabolome captures the downstream functional consequences of gut dysbiosis, thereby serving as the superior and more robust readout for clinical classification.

Consistent with this robust functional readout, our profiling identified distinct molecular signatures associated with MDD pathophysiology. In line with the monoamine hypothesis of depression [29, 30], we observed significantly reduced levels of serotonin and its amino acid precursors (tryptophan, phenylalanine) in the plasma of MDD patients. The depletion of these neurotransmitter precursors suggests a systemic downregulation of biosynthetic pathways critical for mood regulation [31]. Furthermore, our UPLC-QTOF-MS analysis highlighted a disruption in one-carbon metabolism and mitochondrial energy production, evidenced by lower levels of betaine, methionine, cystine, and creatinine [32-34]. The concurrent reduction in urate, a potent antioxidant [35], further supports the hypothesis that MDD is associated with elevated oxidative stress and compromised neuroprotective mechanisms [36].

Beyond isolated metabolic changes, our study highlights the functional connectivity between the gut microbiome and host lipid metabolism. The enrichment of the *E. eligens* group in MDD patients was strongly negatively correlated with plasma stearoyl carnitine, a long-chain acylcarnitine essential for mitochondrial β-oxidation. Since carnitine deficiency is a known feature of depression [27], this suggests that *E. eligens* proliferation may contribute to, or reflect, a maladaptive shift in host energy homeostasis. Conversely, *Veillonella* showed positive associations with propionylcarnitine, likely reflecting its known capacity to produce propionate from lactate [28]. Since propionylcarnitine is a byproduct of propionate metabolism entering the mitochondrial TCA cycle, this correlation suggests that *Veillonella* overgrowth may increase the systemic flux of short-chain fatty acids, thereby altering mitochondrial energy substrates. These associations provide direct evidence that gut dysbiosis in MDD is not merely a co-occurring phenomenon but is functionally intertwined with systemic lipid dysregulation.

Interestingly, while our correlation analysis linked *Veillonella* to specific plasma metabolites, incorporating this taxon into the diagnostic model did not enhance predictive accuracy compared to plasma markers alone. This supports the concept of functional redundancy, where the circulating metabolome acts as a functional readout of microbial activity [37]. Since *Veillonella* is a primary producer of propionate precursors [28], its biological influence is likely fully encapsulated by the observed shifts in plasma propionylcarnitine. Therefore, for clinical purposes, measuring the downstream metabolic effect (carnitines) appears sufficient, rendering the upstream microbial data diagnostically redundant in this specific context.

A key finding of our study was the superior diagnostic performance of the plasma metabolic profile (AUC = 0.862) compared to the gut microbiota composition (AUC = 0.654). This finding aligns with recent systematic reviews indicating that microbiome-based models often yield variable diagnostic accuracy, with performance frequently dropping in validation cohorts due to high inter-individual heterogeneity [38], plasma metabolomics consistently demonstrates higher sensitivity and specificity for psychiatric screening [39, 40]. This discrepancy likely stems from the fundamental physiological differences between these two biological compartments. The gut microbiome is a highly dynamic ecosystem, subject to substantial daily fluctuations driven by diet, medication, and environmental exposures [41]. Consequently, taxonomic signatures based on 16S rRNA sequencing often reflect transient environmental inputs rather than the stable pathophysiological state of the host. Moreover, unlike current clinical diagnostic methods which rely heavily on subjective patient reporting and psychometric scales (*e.g.*, DSM-5 criteria, PHQ-9), this plasma metabolic profile offers a stable, objective biological readout that could complement traditional psychiatric assessments. Notably, integrating gut microbial features into this model yielded only a negligible improvement in diagnostic performance (AUC increase from 0.862 to 0.865), reinforcing the conclusion that the plasma metabolome alone captures the robust diagnostic signal necessary for clinical screening.

In contrast, the plasma metabolome represents the functional 'readout' of the host-microbiome interaction. Before entering systemic circulation, microbial products undergo filtration and modification by the host liver and intestinal barrier—a process that buffers environmental noise and maintains homeostasis [42]. Therefore, circulating metabolites represent a more integrated and stable signal [37, 43]. Furthermore, because MDD is a systemic disorder involving neuroinflammation and neurotransmitter dysregulation [44], plasma metabolites are physiologically proximal to the disease phenotype than the distal gut microbiota [45]. Our data supports the emerging consensus that while the gut microbiome may act as a potential source of bioactive signals, while the circulating metabolome reflects the downstream physiological state associated with these microbial shifts, making it a more robust source of diagnostic biomarkers.

A primary strength of this study lies in its comprehensive multi-platform integration. While previous investigations have often been limited to single-matrix (feces only) or single-platform (GC-MS or LC-MS only) analyses, our study combined 16S rRNA gene sequencing with dual-platform metabolomics (GC-MS and UPLC-QTOF-MS) across both urine and plasma. This 'triangulation' of data allowed us to overcome the coverage limitations of individual techniques—capturing water-soluble primary metabolites, complex lipids, and microbial taxa simultaneously. This holistic approach provided a clearer view of the 'source-to-effector' relationship in MDD pathophysiology than could be achieved by analyzing any single dataset in isolation. Furthermore, this study provides critical multi-omics data specifically for the Korean population. Given that gut microbiome composition is strongly influenced by geography and ethnicity [46], our findings offer a population-specific reference that complements the predominantly Western-centric literature on the gut-brain axis in MDD.

Despite these insights, our study has several limitations that warrant consideration. First, the gut microbiome profiling was based on 16S rRNA gene sequencing, which provides resolution only to the genus level. Consequently, we could not identify specific species or strains of *Veillonella* or *Eubacterium* that might underlie the observed metabolic associations. Second, the sample size was relatively small (*n* = 69) and unbalanced between HC (*n* = 42) and MDD (*n* = 27) groups, which may limit the statistical power and generalizability of our findings. Third, while the age ranges of the MDD and control groups overlapped significantly (32–70 years vs. 21–70 years), the MDD group was older on average. Although we applied rigorous FDR correction to control for multiple testing, we acknowledge that age-related physiological changes could potentially influence metabolic and microbial profiles. Fourth, although we excluded participants with recent antibiotic use, we could not strictly control dietary intake. Diet is known to rapidly alter microbial community structure within 24 h [41], and psychotropic medications can exert direct antimicrobial effects [47]. Therefore, the lack of strict dietary standardization may have introduced variability that obscured more subtle microbial signals. Future studies using longitudinal designs with strictly matched cohorts and dietary controls are needed to validate the stability of these candidate markers.

This study employed an integrated multi-omics approach to characterize the complex interplay between the gut microbiota and systemic metabolism in a Korean cohort with MDD. By combining 16S rRNA gene sequencing with dual-platform metabolomics, we moved beyond isolated observations to reconstruct the functional landscape of the disease. Our results identified specific taxonomic shifts, notably the enrichment of the *E. eligens* group and *Veillonella*, which were intrinsically linked to systemic metabolic disruptions. Specifically, correlation analysis revealed a functional “gut-lipid axis”, where the abundance of these specific taxa was tightly associated with alterations in host fatty acid transport and mitochondrial energy metabolism.

Critically, our diagnostic evaluation demonstrated that while the gut microbiome provides mechanistic insight, the plasma metabolome serves as a superior diagnostic readout. The integrated analysis revealed that the diagnostic information provided by specific gut taxa is largely redundant to the systemic metabolic signals they generate, suggesting that circulating metabolites offer a more stable and proximal biomarker for clinical screening. Ultimately, this study underscores the importance of integrated bioinformatics in psychiatric research. By triangulating data from the gut, urine, and plasma, we have not only characterized potential functional associations between gut dysbiosis to systemic physiology but also established a robust, objective metabolic panel for the precise diagnosis of MDD.

## Supplemental Materials

Supplementary data for this paper are available on-line only at http://jmb.or.kr.



## References

[1] American Psychiatric Association. 2013. *Diagnostic and Statistical Manual of Mental Disorders*. 5th ed. American Psychiatric Association, Washington, D.C. 10.1176/appi.books.9780890425596

[2] Peixia Shi, Aigang Yang, Qing Zhao, Zhaohua Chen, Xiaomei Ren, Qin Dai. 2021. A hypothesis of gender differences in self-reporting symptom of depression: implications to solve under-diagnosis and under-treatment of depression in males. *Front. Psychiatry* **12:** 589687. https://doi.org/10.3389/fpsyt.2021.589687. 10.3389/fpsyt.2021.589687 34759845 PMC8572815

[3] World Health Organization. 2023. Depressive disorder (depression). Available from: https://www.who.int/news-room/fact-sheets/detail/depression. Accessed Nov. 28, 2025.

[4] GBD 2019 Diseases and Injuries Collaborators. 2020. Global burden of 369 diseases and injuries in 204 countries and territories, 1990-2019: a systematic analysis for the Global Burden of Disease Study 2019. *Lancet* **396:** 1204-1222. https://doi.org/10.1016/s0140-6736(20)30925-9. 10.1016/S0140-6736(20)30925-9 33069326 PMC7567026

[5] National Institute of Mental Health. 2023. Major Depression. Available from: https://www.nimh.nih.gov/health/statistics/major-depression. Accessed Dec. 1, 2025.

[6] Léon C de R, Enguerrand R, Beck F. 2023. Prévalence des épisodes dépressifs en France chez les 18-85 ans : résultats du Baromètre santé 2021. *Bull. Epidémiol. Hebd.* **23:** 28-40.

[7] Wang Y, Zhu J, Zhou J. 2025. Disease burden and attributable risk factors of major depressive disorder in China, Japan, and South Korea from 1990 to 2021 and its prediction to 2035. *Front. Public Health* **13:** 1510091. https://doi.org/10.3389/fpubh.2025.1510091. 10.3389/fpubh.2025.1510091 40297030 PMC12034540

[8] Otte C, *et al*. 2016. Major depressive disorder. *Nature Reviews Disease Primers* **2:** 1-20. 10.1038/nrdp.2016.65 27629598

[9] Rudolf Uher, Jennifer L Payne, Barbara Pavlova, Roy H Perlis. 2014. Major depressive disorder in DSM-5: implications for clinical practice and research of changes from DSM-IV. *Depres. Anxiety* **31:** 459-471. https://doi.org/10.1002/da.22217. 10.1002/da.22217 24272961

[10] Karissa M Johnston, Lauren C Powell, Ian M Anderson, Shelagh Szabo, Stephanie Cline. 2019. The burden of treatment-resistant depression: a systematic review of the economic and quality of life literature. *J. Affect. Disord.* **242:** 195-210. https://doi.org/10.1016/j.jad.2018.06.045. 10.1016/j.jad.2018.06.045 30195173

[11] John F Cryan, Kenneth J O'Riordan, Caitlin S M Cowan, Kiran V Sandhu, Thomaz F S Bastiaanssen, Marcus Boehme, *et al*. 2019. The microbiota-gut-brain axis. *Physiol. Rev.* **99:** 1877-2013. 10.1152/physrev.00018.2018 31460832

[12] Chun-Hua Zhou, Yu-Ting Meng, Jia-Jia Xu, Xue Fang, Jiu-Long Zhao, Wei Zhou, *et al*. 2020. Altered diversity and composition of gut microbiota in Chinese patients with chronic pancreatitis. *Pancreatology* **20:** 16-24. https://doi.org/10.1016/j.pan.2019.11.013. 10.1016/j.pan.2019.11.013 31806503

[13] Zaiquan Dong, Xiaoling Shen, Yanni Hao, Jin Li, Haoran Li, Haizheng Xu, *et al*. 2021. Gut microbiome: a potential indicator for differential diagnosis of major depressive disorder and general anxiety disorder. *Front. Psychiatry* **12:** 651536. 10.3389/fpsyt.2021.651536 34589003 PMC8473618

[14] Rick Jansen, Yuri Milaneschi, Daniela Schranner, Gabi Kastenmuller, Matthias Arnold, Xianlin Han, *et al*. 2024. The metabolome-wide signature of major depressive disorder. *Mol. Psychiatry* **29:** 3722-3733. https://doi.org/10.1038/s41380-024-02613-6. 10.1038/s41380-024-02613-6 38849517

[15] Peng Zheng, Ying Wang, Liang Chen, Deyu Yang, Huaqing Meng, Dezhi Zhou, *et al*. 2013. Identification and validation of urinary metabolite biomarkers for major depressive disorder. *Mol. Cell. Proteomics* **12:** 207-214. https://doi.org/10.1074/mcp.m112.021816. 10.1074/mcp.M112.021816 23111923 PMC3536901

[16] Erik von Elm, Douglas G Altman, Matthias Egger, Stuart J Pocock, Peter C Gøtzsche, Jan P Vandenbroucke, *et al*. 2007. The Strengthening the Reporting of Observational Studies in Epidemiology (STROBE) statement: guidelines for reporting observational studies. *Lancet* **370:** 1453-1457. https://doi.org/10.1016/s0140-6736(07)61602-x. 10.1016/S0140-6736(07)61602-X 18064739

[17] Benjamin J Callahan, Paul J McMurdie, Michael J Rosen, Andrew W Han, Amy Jo A Johnson, Susan P Holmes. 2016. DADA2: high-resolution sample inference from Illumina amplicon data. *Nat. Methods* **13:** 581-583. https://doi.org/10.1038/nmeth.3869. 10.1038/nmeth.3869 27214047 PMC4927377

[18] Evan Bolyen, Jai Ram Rideout, Matthew R Dillon, Nicholas A Bokulich, Christian C. Abnet, Gabriel A Al-Ghalith, *et al*. 2019. Reproducible, interactive, scalable and extensible microbiome data science using QIIME 2. *Nat. Biotechnol.* **37:** 852-857. 10.1038/s41587-019-0209-9 PMC7015180 31341288

[19] Christian Quast, Elmar Pruesse, Pelin Yilmaz, Jan Gerken, Timmy Schweer, Pablo Yarza, *et al*. 2012. The SILVA ribosomal RNA gene database project: improved data processing and web-based tools. *Nucleic Acids Res.* **41(D1):** D590-D596. https://doi.org/10.1093/nar/gks1219. 10.1093/nar/gks1219 23193283 PMC3531112

[20] Oksanen J, *et al*. 2013. Package 'vegan'. *Community Ecology Package* **2:** 1-295.

[21] Paradis E, Claude J, Strimmer K. 2004. APE: analyses of phylogenetics and evolution in R language. *Bioinformatics* **20:** 289-290. https://doi.org/10.1093/bioinformatics/btg412. 10.1093/bioinformatics/btg412 14734327

[22] Wickham H. 2016. Getting Started with ggplot2, pp. 11-31. In Wickham H (ed.), *ggplot2: Elegant Graphics for Data Analysis*. Springer, New York. 10.1007/978-3-319-24277-4_2

[23] Jungyeon Kim, Joong Kyong Ahn, Yu Eun Cheong, Sung-Joon Lee, Hoon-Suk Cha, Kyoung Heon Kim. 2020. Systematic re-evaluation of the long-used standard protocol of urease-dependent metabolome sample preparation. *PLoS One* **15:** e0230072. https://doi.org/10.1371/journal.pone.0230072. 10.1371/journal.pone.0230072 32182259 PMC7077817

[24] Hiroshi Tsugawa, Tomas Cajka, Tobias Kind, Yan Ma, Brendan Higgins, Kazutaka Ikeda, *et al*. 2015. MS-DIAL: data-independent MS/MS deconvolution for comprehensive metabolome analysis. *Nature Methods* **12:** 523-526. https://doi.org/10.1038/nmeth.3393. 10.1038/nmeth.3393 25938372 PMC4449330

[25] Mariana Millan Fachi, Letícia Bonancio Cerqueira, Letícia Paula Leonart, Thais Martins Guimarães de Francisco, Roberto Pontarolo. 2016. Simultaneous quantification of antidiabetic agents in human plasma by a UPLC-QToF-MS method. *PLoS One* **11:** e0167107. https://doi.org/10.1371/journal.pone.0167107. 10.1371/journal.pone.0167107 27930700 PMC5145167

[26] Xavier Robin, Natacha Turck, Alexandre Hainard, Natalia Tiberti, Frédérique Lisacek, Jean-Charles Sanchez, *et al*. 2011. pROC: an open-source package for R and S+ to analyze and compare ROC curves. *BMC Bioinformatics* **12:** 77. https://doi.org/10.1186/1471-2105-12-77. 10.1186/1471-2105-12-77 21414208 PMC3068975

[27] Carla Nasca, Benedetta Bigio, Francis S Lee, Sarah P Young, Marin M Kautz, Ashly Albright, *et al*. 2018. Acetyl-L-carnitine deficiency in patients with major depressive disorder. *Proc. Natl. Acad. Sci. USA* **115:** 8627-8632. https://doi.org/10.1073/pnas.1801609115. 10.1073/pnas.1801609115 30061399 PMC6112703

[28] Jonathan Scheiman, Jacob M Luber, Theodore A Chavkin, Tara MacDonald, Angela Tung, Loc-Duyen Pham, *et al*. 2019. Meta-omics analysis of elite athletes identifies a performance-enhancing microbe that functions via lactate metabolism. *Nat. Med.* **25:** 1104-1109. https://doi.org/10.1038/s41591-019-0485-4. 10.1038/s41591-019-0485-4 31235964 PMC7368972

[29] Hirschfeld RM. 2000. History and evolution of the monoamine hypothesis of depression. *J. Clin. Psychiatry* **61:** 4-6. 10775017

[30] Pastis I, Santos MG, Paruchuri A. 2024. Exploring the role of inflammation in major depressive disorder: beyond the monoamine hypothesis. *Front. Behav. Neurosci.* **17:** 1282242. https://doi.org/10.3389/fnbeh.2023.1282242. 10.3389/fnbeh.2023.1282242 38299049 PMC10829100

[31] Shintaro Ogawa, Takashi Fujii, Norie Koga, Hiroaki Hori, Toshiya Teraishi, Kotaro Hattori, *et al*. 2014. Plasma L-tryptophan concentration in major depressive disorder: new data and meta-analysis. *J. Clin. Psychiatry* **75:** e906-e915. https://doi.org/10.4088/jcp.13r08908. 10.4088/JCP.13r08908 25295433

[32] Allen PJ. 2012. Creatine metabolism and psychiatric disorders: does creatine supplementation have therapeutic value? *Neurosci. Biobehav. Rev.* **36:** 1442-1462. https://doi.org/10.1016/j.neubiorev.2012.03.005. 10.1016/j.neubiorev.2012.03.005 22465051 PMC3340488

[33] Rongji Hui, Jiabao Xu, Hongchen Ma, Tao Feng, Congcong Hou, Xintao Wang, *et al*. 2025. Disrupted betaine metabolism drives Th17 cell differentiation, mediating methamphetamine-induced depressive behaviors in male mice. *J. Neuroinflammation* **22:** 207. https://doi.org/10.1186/s12974-025-03532-1. 10.1186/s12974-025-03532-1 40859333 PMC12379426

[34] P Zheng, B Zeng, C Zhou, M Liu, Z Fang, X Xu, *et al*. 2016. Gut microbiome remodeling induces depressive-like behaviors through a pathway mediated by the host's metabolism. *Mol. Psychiatry* **21:** 786-796. https://doi.org/10.1038/mp.2016.44. 10.1038/mp.2016.44 27067014

[35] Catherine N Black, Mariska Bot, Peter G Scheffer, Harold Snieder, Brenda W J H Penninx. 2018. Uric acid in major depressive and anxiety disorders. *J. Affect. Disord.* **225:** 684-690. https://doi.org/10.1016/j.jad.2017.09.003. 10.1016/j.jad.2017.09.003 28917195

[36] Steven Moylan, Michael Berk, Olivia M Dean, Yuval Samuni, Lana J Williams, Adrienne O'Neil, *et al*. 2014. Oxidative & nitrosative stress in depression: why so much stress? *Neurosci. Biobehav. Rev.* **45:** 46-62. https://doi.org/10.1016/j.neubiorev.2014.05.007. 10.1016/j.neubiorev.2014.05.007 24858007

[37] Alessia Visconti, Caroline I Le Roy, Fabio Rosa, Niccolò Rossi, Tiphaine C Martin, Robert P Mohney, *et al*. 2019. Interplay between the human gut microbiome and host metabolism. *Nat. Commun.* **10:** 4505. https://doi.org/10.1038/s41467-019-12476-z. 10.1038/s41467-019-12476-z 31582752 PMC6776654

[38] Djawad Radjabzadeh, Cindy G Boer, Sanne A Beth, Pelle van der Wal, Jessica C Kiefte-De Jong, Michelle A E Jansen, *et al*. 2020. Diversity, compositional and functional differences between gut microbiota of children and adults. *Sci. Rep.* **10:** 1040. https://doi.org/10.1038/s41598-020-57734-z. 10.1038/s41598-020-57734-z 31974429 PMC6978381

[39] Kellie MacDonald, Ankur Krishnan, Emily Cervenka, Grace Hu, Elena Guadagno, Yannis Trakadis . 2019. Biomarkers for major depressive and bipolar disorders using metabolomics: a systematic review. *Am. J. Med. Genet. B Neuropsychiatr. Genet.* **180:** 122-137. https://doi.org/10.1002/ajmg.b.32680. 10.1002/ajmg.b.32680 30411484

[40] Jun-Xi Pan, Jin-Jun Xia, Feng-Li Deng, Wei-Wei Liang, Jing Wu, Bang-Min Yin, *et al*. 2018. Diagnosis of major depressive disorder based on changes in multiple plasma neurotransmitters: a targeted metabolomics study. *Transl. Psychiatry* **8:** 130. https://doi.org/10.1038/s41398-018-0183-x. 10.1038/s41398-018-0183-x 29991685 PMC6039504

[41] Lawrence A David, Corinne F Maurice, Rachel N Carmody, David B Gootenberg, Julie E Button, Benjamin E Wolfe, *et al*. 2014. Diet rapidly and reproducibly alters the human gut microbiome. *Nature* **505:** 559-563. 10.1038/nature12820 24336217 PMC3957428

[42] Maria L Balmer, Emma Slack, Andrea de Gottardi, Melissa A E Lawson, Siegfried Hapfelmeier, Luca Miele , *et al*. 2014. The liver may act as a firewall mediating mutualism between the host and its gut commensal microbiota. *Sci. Transl. Med.* **6:** 237ra66. https://doi.org/10.1126/scitranslmed.3008618. 10.1126/scitranslmed.3008618 24848256

[43] Christian Diener, Chengzhen L Dai, Tomasz Wilmanski, Priyanka Baloni, Brett Smith, Noa Rappaport, *et al*. 2022. Genome-microbiome interplay provides insight into the determinants of the human blood metabolome. *Nat. Metab.* **4:** 1560-1572. https://doi.org/10.1038/s42255-022-00670-1. 10.1038/s42255-022-00670-1 36357685 PMC9691620

[44] Miller AH, Raison CL. 2016. The role of inflammation in depression: from evolutionary imperative to modern treatment target. *Nat. Rev. Immunol.* **16:** 22-34. https://doi.org/10.1038/nri.2015.5. 10.1038/nri.2015.5 26711676 PMC5542678

[45] Patti GJ, Yanes O, Siuzdak G. 2012. Metabolomics: the apogee of the omics trilogy. *Nat. Rev. Mol. Cell Biol.* **13:** 263-269. 10.1038/nrm3314 22436749 PMC3682684

[46] Yan He, Wei Wu, Hui-Min Zheng, Pan Li, Daniel McDonald, Hua-Fang Sheng, *et al*. 2018. Regional variation limits applications of healthy gut microbiome reference ranges and disease models. *Nat. Med.* **24:** 1532-1535. https://doi.org/10.1038/s41591-018-0164-x. 10.1038/s41591-018-0164-x 30150716

[47] Lisa Maier, Mihaela Pruteanu, Michael Kuhn, Georg Zeller, Anja Telzerow, Exene Erin Anderson, *et al*. 2018. Extensive impact of non-antibiotic drugs on human gut bacteria. *Nature* **555:** 623-628. https://doi.org/10.1038/nature25979. 10.1038/nature25979 29555994 PMC6108420

