# Supplementary material for: Integrated Microbiome and Metabolomic Profiling to Identify Potential Biomarkers of Major Depressive Disorder
Source: J Microbiol Biotechnol. 2026 Jan 22;36:e2512014. doi: 10.4014/jmb.2512.12014 (PMC12868950; doi:10.4014/jmb.2512.12014)
Supplement: Supplementary file 1 [file jmb-36-e2512014-supple.pdf]

J. Microbiol. Biotechnol. 2026. 36: e2512014

https://doi.org/10.4014/jmb.2512.12014

Supplementary Tables

Table S1. Korean Beck Depression Inventory-II (K-BDI-II). A self-report questionnaire used to assess the severity of depression.

Korean - Beck Depression Inventory - II

Instructions: This questionnaire consists of 21 items. Please read the sentences presented in each item carefully and circle the number of the one sentence that best expresses how you have felt for **the past two weeks, including today**. If multiple sentences in one item apply to you equally, choose the sentence with the highest score for each item. Please be sure to select only one sentence, including item 16 (Changes in Sleep Pattern) and item 18 (Changes in Appetite).

1. Sadness
0 I do not feel sad.
1 I feel sad much of the time.
2 I am sad all the time.
3 I am so sad or unhappy that I can't stand it.

2. Pessimism
0 I am not discouraged about my future.
1 I feel more discouraged about my future than I used to be.
2 I do not expect things to work out for me.
3 I feel my future is hopeless and will only get worse.

3. Past Failure
0 I do not feel like a failure.
1 I have failed more than I thought.
2 As I look back, I see a lot of failures.
3 I feel I am a total failure as a person.

4. Loss of Pleasure
0 I get as much pleasure as I ever did from the things I enjoy.
1 I don't enjoy things as much as I used to.
2 I get very little pleasure from the things I used to enjoy.
3 I get no pleasure from the things I used to enjoy.

5. Guilty Feelings
0 I don't feel particularly guilty.
1 I feel guilty over many things I have done or should have done.
2 I feel guilty much of the time.
3 I feel guilty all of the time.

6. Punishment Feelings
0 I don't feel I am being punished.
1 I feel I may be punished.
2 I expect to be punished.
3 I feel I am being punished.

7. Self-Dislike
0 I feel the same about myself as ever.
1 I have lost confidence in myself.
2 I am disappointed in myself.
3 I dislike myself.

8. Self-Criticism
0 I don't criticize or blame myself more than usual.
1 I am more critical of myself than I used to be.
2 I criticize myself for all my faults.
3 I blame myself for everything bad that happens.

9. Suicidal Thoughts or Wishes
0 I don't have any thoughts of killing myself.
1 I have thoughts of killing myself, but I would not carry them out.
2 I would like to kill myself.
3 I would kill myself if I had the chance.

10. Crying
0 I don't cry any more than I used to.
1 I cry more than I used to.
2 I cry over every little thing.
3 I feel like crying, but I can't.

11. Agitation
0 I am no more restless or wound up than usual.
1 I feel more restless or wound up than usual.
2 I am so restless or agitated that it's hard to stay still.
3 I am so restless or agitated that I have to keep moving or doing something.

12. Loss of Interest
0 I have not lost interest in other people or activities.
1 I am less interested in other people or things than I used to be.
2 I have lost most of my interest in other people or things.
3 It's hard for me to get interested in anything.

13. Indecisiveness
0 I make decisions about as well as ever.
1 I find it more difficult to make decisions than I used to.
2 I have much greater difficulty in making decisions than I used to.
3 I have trouble making any decisions.

14. Worthlessness
0 I do not feel I am worthless.
1 I don't feel I am as worthwhile and useful as I used to be.
2 I feel more worthless than other people.
3 I feel utterly worthless.

15. Loss of Energy
0 I have as much energy as ever.
1 I have less energy than I used to have.
2 I have a lot less energy.
3 I don't have enough energy to do anything.

16. Changes in Sleeping Pattern
0 I have not experienced any change in my sleeping pattern.
1a I sleep slightly more than usual.
1b I sleep slightly less than usual.
2a I sleep much more than usual.
2b I sleep much less than usual.
3a I sleep most of the day.
3b I wake up 1-2 hours earlier than usual and find it hard to get back to sleep.

17. Irritability
0 I am no more irritable than usual.
1 I am more irritable than usual.
2 I am much more irritable than usual.
3 I am irritable all the time.

18. Changes in Appetite
0 I have not experienced any change in my appetite.
1a My appetite is slightly less than usual.
1b My appetite is slightly greater than usual.
2a My appetite is much less than usual.
2b My appetite is much greater than usual.
3a I have no appetite at all.
3b I crave food all the time.

19. Concentration Difficulty
0 I can concentrate as well as ever.
1 I can't concentrate as well as usual.
2 It's hard for me to concentrate on anything for a long time.
3 I find I can't concentrate on anything.

20. Tiredness or Fatigue
0 I am no more tired or fatigued than usual.
1 I get tired or fatigued more easily than usual.
2 I am too tired or fatigued to do many of the things I used to do.
3 I am too tired or fatigued to do any of the things I used to do.

21. Loss of Interest in Sex
0 I have not noticed any recent change in my interest in sex.
1 I am less interested in sex than I used to be.
2 I am much less interested in sex now.
3 I have lost interest in sex completely.

Table S2. DSM-5 Criteria for Major Depressive Disorder (MDD). Official diagnostic guidelines used by clinicians to identify Major Depressive Disorder.

DSM-5 Diagnosis for Major Depressive Disorder (MDD)

At least one of the symptoms - (1) depressed mood or (2) loss of interest or pleasure – must be present, and five or more of the following symptoms have been present during the same 2-week period.

1. Depressed mood most of the day, nearly every day, as indicated by either subjective report (e.g., feels sad, empty, hopeless) or observation made by others (e.g., appears tearful).
2. Markedly diminished interest or pleasure in all, or almost all, activities most of the day, nearly every day.
3. Significant weight loss or gain or decrease or increase in appetite.
4. Insomnia or hypersomnia nearly every day.
5. Psychomotor agitation (e.g., restlessness) or retardation (e.g., slowed thoughts or actions) nearly every day.
6. Fatigue or loss of energy nearly every day.
7. Feelings of worthlessness or excessive or inappropriate guilt nearly every day.
8. Diminished ability to think or concentrate, or indecisiveness, nearly every day.
9. Recurrent thoughts of death, recurrent suicidal ideation without a specific plan, or a suicide attempt or a specific plan for committing suicide.

The symptoms cause clinically significant distress or impairment in social, occupational, or other important areas of functioning.
The disturbance is not attributable to the physiological effects of a substance (e.g., drugs, medication) or another medical condition.

Table S3. Characteristics of participants in the healthy and MDD groups.

	HC (n=42)	MDD (n=27)	*p*-value
**Age (range)**	42.71 ± 16.20 (21-70)	58.63 ± 11.94 (31-70)	0.001
**Sex (Male / Female)**	12 (28.57%) / 30 (71.43%)	6 (22.22%) / 21 (77.78%)	0.56

Table S4. Summary of statistical testing results for gut microbial taxa at the genus level between HC and MDD groups. The table presents raw *p*-values (Welch's t-test or Mann–Whitney U test), FDR-adjusted *q*-values, and effect sizes (Hedges' *g*).

No.	Genus	*p*-value	*q*-value	Hedges' *g*
1	Lachnospiraceae	0.5340	0.7992	0.3040
2	*Eubacterium coprostanoligenes* group	0.3359	0.7630	0.2328
3	*Eubacterium eligens* group	0.0029	0.0744	-0.6433
4	*Eubacterium ruminantium* group	0.2386	0.6954	0.2361
5	*Eubacterium siraeum* group	0.3519	0.7630	0.0595
6	*Ruminococcus torques* group	0.6621	0.8563	0.1340
7	*Agathobacter*	0.7591	0.8563	0.0640
8	*Akkermansia*	0.7849	0.8563	-0.0138
9	*Alistipes*	0.5661	0.7992	-0.1809
10	*Alloprevotella*	0.2463	0.6954	-0.0789
11	*Anaerostipes*	0.2080	0.6954	-0.5126
12	*Bacteroides*	0.4779	0.7923	-0.1777
13	*Blautia*	0.3495	0.7630	-0.4693
14	*Butyrivibrio*	0.2208	0.6954	-0.4316
15	CAG-352	0.7231	0.8563	0.0682
16	Christensenellaceae R-7 group	0.3380	0.7630	-0.2589
17	Clostridia UCG-014	0.8689	0.9067	0.1672
18	*Clostridium* sensu stricto 1	0.0319	0.5104	-0.5638
19	*Coprococcus*	0.1674	0.6954	0.3198
20	*Dialister*	0.1495	0.6954	0.1911
21	*Eisenbergiella*	0.3815	0.7630	0.2633
22	*Enterobacter*	0.3687	0.7630	-0.3317
23	*Escherichia-Shigella*	0.2031	0.6954	-0.0675
24	*Faecalibacterium*	0.1202	0.6954	-0.4186
25	*Fusicatenibacter*	0.2735	0.7293	0.2827
26	*Klebsiella*	0.7669	0.8563	-0.2840
27	Lachnoclostridium	0.0948	0.6954	0.3314
28	Lachnospira	0.7249	0.8563	-0.2633
29	Lachnospiraceae ND3007 group	0.4719	0.7923	0.0323
30	Lachnospiraceae NK4A136 group	0.6259	0.8441	-0.3003
31	Lachnospiraceae UCG-004	0.6331	0.8441	-0.2071

No.	Genus	*p*-value	*q*-value	Hedges' *g*
32	NK4A214 group	0.8479	0.9044	0.0343
33	*Odoribacter*	0.9895	0.9895	-0.2393
34	*Oscillibacter*	0.2232	0.6954	0.2248
35	*Parabacteroides*	0.5657	0.7992	-0.2590
36	*Paraprevotella*	0.2267	0.6954	-0.4652
37	*Parasutterella*	0.7841	0.8563	-0.1485
38	*Phascolarctobacterium*	0.4952	0.7923	0.0261
39	*Prevotella*	0.3993	0.7667	0.2917
40	*Prevotella* 9	0.1014	0.6954	0.4079
41	*Roseburia*	0.5152	0.7977	-0.1744
42	*Ruminococcus*	0.0535	0.6420	-0.5860
43	*Streptococcus*	0.8972	0.9163	0.0358
44	*Subdoligranulum*	0.4633	0.7923	-0.2839
45	*Sutterella*	0.4814	0.7923	0.2111
46	UCG-002	0.2279	0.6954	0.4064
47	UCG-003	0.7330	0.8563	0.0278
48	*Veillonella*	0.0031	0.0744	-0.3640

Table S5. List of metabolites identified in urine and plasma samples with a VIP > 1.0 using UPLC-QTOF-MS and GC-MS. Metabolites with a q < 0.05 are highlighted.

No.	Urine Metabolite (GC-MS)	Plasma Metabolite (GC-MS)	Plasma Metabolite (UPLC-QTOF-MS)
1	1,5-Anhydroglucitol	Alanine	(-)-Stachydrine
2	1,6-Anhydroglucose	Glutamine	(2R,3R,4R,6aR,6bS,8aR,14bR)-2,3,12-trihydroxy-4,6a,6b,11,11,14b-hexamethyl-8a-[(2S,3R,4S,5S,6R)-3,4,5-trihydroxy-6-(hydroxymethyl)oxan-2-yl]oxycarbonyl-1,2,3,4a,5,6,7,8,9,10,12,12a,14,14a-tetradecahydropicene-4-carboxylic acid
3	1-Deoxyerythritol	Glyceric acid	1-Heptadecanoyl-sn-glycero-3-phosphocholine
4	1-Methylgalactose	Inositol	1-LysoPC(18:2(c9,t12))
5	3,4-Dihydrophenylacetic acid	Isoleucine	2-o-Methylcytidine
6	3-Hydroxyisovaleric acid	Leucine	2-palmitoyl-sn-glycero-3-phosphocholine
7	Alanine	Lysine	3,5-dihydroxydecanoic acid
8	Ascorbic acid	Methionine	3-Indoleacrylic acid
9	Cystine	Palmitic acid	5-Aminovaleric acid
10	Ferulic acid	Phenylalanine	Aminocaproic acid
11	Fructose	Proline	Benzoic Acid
12	Galactitol	Propyleneglycol	Betaine
13	Galactose	Serine	Creatine
14	Glucose	Stearic acid	Creatinine
15	Glycerol	Threonic acid	Deoxyribose 5-phosphate
16	Glycolic acid	Threonine	Docosahexaenoic acid
17	Histidine	Urea	FA 18:1+1O
18	Hypoxanthine	Valine	Glycochenodeoxycholic Acid
19	Inositol		Glycochenodeoxycholate
20	Lysine		Heptadecanoic Acid
21	N-Acetyl-L-aspartic acid		Heptanoylcarnitine
22	O-Phosphoethanolamine		Leucylproline
23	Oxalic acid		L-Hexanoylcarnitine
24	Phenoxyacetic acid		L-Phenylalanine
25	Pyruvate		L-Proline
26	Quinic acid		L-Pyroglutamic Acid
27	Serine		LysoPC(0:0/18:0)
28	Sucrose		LysoPC(16:0/0:0)
29	Tagatose		LysoPC(16:1)
30	Tartaric acid		LysoPC(18:0/0:0)

31	Tyramine	LysoPC(18:1/0:0)
32	Uric acid	LysoPC(18:2/0:0)
33		LysoPC(20:1/0:0)
34		LysoPE(0:0/18:3)
35		LysoPE(16:0/0:0)
36		MGMG 18:2
37		Nicotinate D-ribonucleotide
38		PC(15:0/0:0)
39		PC(15:0/0:0)
40		Propionylcarnitine
41		PS(17:0/20:4)
42		Stearoyl Carnitine
43		Thymidine
44		Urate
45		Urea
46		Uric acid

Table S6. Summary of statistical testing results for GC-MS urinary metabolites between the HC and MDD groups. The table presents raw *p*-values (Welch's t-test or Mann–Whitney U test), FDR-adjusted *q*-values, and effect sizes (Hedges' *g*)

No.	Metabolite	*p*-value	*q*-value	Hedges' *g*
1	1,5-Anhydroglucitol	0.8236	0.9179	0.3056
2	1,6-Anhydroglucose	0.7118	0.8844	-0.3876
3	1-Deoxyerythritol	0.0382	0.4474	-0.5815
4	1-Methylgalactose	0.4577	0.7478	-0.3797
5	2-Aminoadipic acid	0.7485	0.9042	-0.0369
6	2-Aminoethanol	0.2841	0.6913	0.2622
7	2-Dehydro-D-gluconate	0.8499	0.9179	0.2098
8	2-Deoxytetronic acid	0.4879	0.7549	0.1480
9	2-Oxoglutaric acid	0.3509	0.6913	0.2343
10	3,4-Dihydrophenylacetic acid	0.1611	0.6913	-0.3812
11	3-Aminoisobutyric acid	0.3493	0.6913	0.1707
12	3-Hydroxy-3-methylglutarate	0.1520	0.6913	-0.0887
13	3-Hydroxyisovaleric acid	0.0835	0.5595	0.3517
14	3-Hydroxyphenylacetic acid	0.9149	0.9496	-0.2455
15	3-Methoxy-4-hydroxymandelate	0.9683	0.9683	0.0243
16	3-Methylglutarate	0.7718	0.9042	-0.0958
17	4-Hydroxyphenylacetic acid	0.6143	0.8211	-0.2882
18	5-Hydroxyindoleactate	0.6802	0.8609	0.2970
19	Alanine	0.0887	0.5595	-0.4116
20	Allose	0.3430	0.6913	-0.2908
21	Ascorbic acid	0.1824	0.6913	-0.4434
22	Benzylalcohol	0.5846	0.8125	-0.2296
23	Butane-2,3-diol	0.8619	0.9179	-0.0967
24	Catechol	0.8956	0.9415	-0.2928
25	Citric acid	0.2897	0.6913	-0.1986
26	Cystine	0.0455	0.4474	0.4592
27	Dehydroascorbic acid	0.0491	0.4474	-0.2016
28	DL-beta-Hydroxybutyric acid	0.8213	0.9179	0.1161
29	Ferulic acid	0.2240	0.6913	-0.3198
30	Fructose	0.6208	0.8211	0.4795
31	Galactitol	0.3953	0.7203	-0.3864

No.	Metabolite	*p*-value	*q*-value	Hedges' *g*
32	Galactose	0.3541	0.6913	-0.3685
33	Glucarate	0.7625	0.9042	-0.2643
34	Gluconic acid	0.3686	0.6961	-0.1582
35	Glucose	0.4448	0.7478	-0.3773
36	Glucuronate	0.8618	0.9179	-0.3078
37	Glyceric acid	0.9440	0.9676	0.0772
38	Glycerol	0.1042	0.6103	0.4396
39	Glycine	0.5617	0.7941	0.2125
40	Glycolic acid	0.0108	0.1804	0.5915
41	Hippuric acid	0.6824	0.8609	0.0914
42	Histidine	0.0284	0.3881	0.3656
43	Hypoxanthine	0.2263	0.6913	0.3883
44	Inositol	0.0018	0.1476	-0.6846
45	Lactose	0.4285	0.7478	-0.2954
46	Leucine	0.3735	0.6961	-0.0045
47	Lysine	0.0058	0.1585	0.3963
48	Malic acid	0.2311	0.6913	-0.3230
49	Mannitol	0.0609	0.4756	0.1841
50	N-Acetyl-L-aspartic acid	0.3322	0.6913	0.3419
51	O-Phosphoethanolamine	0.2125	0.6913	-0.4151
52	Oxalic acid	0.4540	0.7478	-0.5175
53	Pantothenate	0.3231	0.6913	-0.2002
54	Phenol	0.4595	0.7478	0.2973
55	Phenoxyacetic acid	0.1950	0.6913	-0.3581
56	Phenylalanine	0.2408	0.6913	0.1271
57	Phosphate	0.5453	0.7941	-0.0754
58	Propyleneglycol	0.5514	0.7941	0.1808
59	Pyrogallol	0.7719	0.9042	-0.3095
60	Pyroglutamic acid	0.5433	0.7941	0.1544
61	Pyruvate	0.2534	0.6913	0.3488
62	Quinic acid	0.4746	0.7484	0.3639
63	Serine	0.9586	0.9683	-0.1832
64	Sorbitol	0.5617	0.7941	-0.1034
65	Sucrose	0.1153	0.6303	-0.4096
66	Sulfuric acid	0.6824	0.8609	-0.1349

No.	Metabolite	*p*-value	*q*-value	Hedges' *g*
67	Tagatose	0.1264	0.6478	0.4988
68	Tartaric acid	0.0048	0.1585	-0.5824
69	Taurine	0.6057	0.8211	0.0583
70	Threitol	0.2182	0.6913	-0.2881
71	Threonic acid	0.2947	0.6913	0.2441
72	Threonine	0.4651	0.7478	0.0034
73	Tyramine	0.0110	0.1804	0.3343
74	Tyrosine	0.1715	0.6913	0.1711
75	Uracil	0.7953	0.9179	0.2008
76	Urea	0.8499	0.9179	-0.2365
77	Uric acid	0.0638	0.4756	0.4449
78	Valine	0.3261	0.6913	0.2146
79	Xylitol	0.3368	0.6913	0.1636
80	Xylonic acid	0.3269	0.6913	-0.2403
81	Xylose	0.1359	0.6555	-0.1115
82	Xylulose	0.3112	0.6913	0.2870

Table S7. Summary of statistical testing results for GC-MS plasma metabolites between the HC and IBS groups. The table presents raw *p*-values (Welch's t-test or Mann–Whitney U test), FDR-adjusted *q*-values, and effect sizes (Hedges' *g*)

No.	Metabolite	*p*-value	*q*-value	Hedges' *g*
1	1,5-Anhydro glucitol	0.0365	0.1794	0.3801
2	2-Hydroxybutyrate	0.9951	0.9951	0.0047
3	Alanine	0.3993	0.6008	0.2076
4	Cholesterol	0.3430	0.6008	0.2922
5	Citric acid	0.6937	0.7957	-0.0351
6	Dihydroxyacetone	0.6936	0.7957	-0.0626
7	DL-beta-Hydroxybutyric acid	0.5211	0.7035	0.2067
8	Dopamine	0.8094	0.9019	-0.1547
9	Elaidic acid	0.9586	0.9838	-0.1711
10	Galactose	0.3886	0.6008	0.3211
11	Glutamic acid	0.3261	0.6008	0.6537
12	Glutamine	0.0087	0.0907	-0.4483
13	Glyceric acid	0.0881	0.2643	0.0357
14	Glycine	0.3493	0.6008	0.1008
15	Glycolic acid	0.9101	0.9669	0.3622
16	Hydroxyproline	0.2967	0.6008	0.2840
17	Hypoxanthine	0.1648	0.4285	-0.5182
18	Inositol	0.0093	0.0907	0.0240
19	Isoleucine	0.0600	0.1950	0.5396
20	Lactic acid	0.5132	0.7035	0.0202
21	Leucine	0.0149	0.1162	0.6578
22	Lysine	0.0505	0.1798	0.4698
23	Methionine	0.0067	0.0907	0.6772
24	Phenylalanine	0.0030	0.0907	0.7929
25	Phosphate	0.9173	0.9669	-0.0727
26	Proline	0.0368	0.1794	0.4730
27	Propyleneglycol	0.4005	0.6008	0.3696
28	Pyroglutamic acid	0.5823	0.7326	-0.1421
29	Serine	0.2561	0.5875	0.2776
30	Sorbitol	0.3768	0.6008	-0.0332
31	Threitol	0.5803	0.7326	0.0340
32	Threonic acid	0.2057	0.5014	-0.2587

No.	Metabolite	*p*-value	*q*-value	Hedges' *g*
33	Threonine	0.0416	0.1798	0.5269
34	Tryptophan	0.0507	0.1798	0.4743
35	Tyrosine	0.1460	0.4067	0.4035
36	Urea	0.2897	0.6008	-0.1507
37	Uric acid	0.6824	0.7957	0.2980
38	Valine	0.0211	0.1372	0.6045
39	Xylose	0.5231	0.7035	-0.2371

Table S8. Summary of statistical testing results for UPLC-QTOF-MS (positive mode) plasma metabolites between the HC and IBS groups. The table presents raw *p*-values (Welch's t-test or Mann–Whitney U test), FDR-adjusted *q*-values, and effect sizes (Hedges' *g*)

No.	Metabolite	*p*-value	*q*-value	Hedges' *g*
1	(-)-Stachydrine	0.6469	0.7276	0.0536
2	(2R,3R,4R,6aR,6bS,8aR,14bR)-2,3,12-trihydroxy-4,6a,6b,11,11,14b-hexamethyl-8a-[(2S,3R,4S,5S,6R)-3,4,5-trihydroxy-6-(hydroxymethyl)oxan-2-yl]oxycarbonyl-1,2,3,4a,5,6,7,8,9,10,12,12a,14,14a-tetradecahydropicene-4-carboxylic acid	0.0001	0.0006	0.8935
3	11,12-dihydroxy-5Z,8Z,14Z-eicosatrienoic acid	0.3368	0.4463	0.1517
4	16-Hydroxyhexadecanoic acid	0.0115	0.0271	-0.6335
5	1-eicosapentaenoyl-2-docosahexaenoyl-sn-glycero-3-phosphoglycerol	0.6181	0.7174	0.0088
6	1-Heptadecanoyl-sn-glycero-3-phosphocholine	0.0021	0.0070	0.7203
7	1-Hexadecyl Lysophosphatidic Acid	0.8494	0.8741	-0.0937
8	1-LysoPC(18:2(c9,t12))	0.0001	0.0006	1.1889
9	1-Methyladenosine	0.2091	0.3078	0.3957
10	2-Hydroxyadipic acid	0.0032	0.0097	-0.7246
11	2-Octenoyl carnitine	0.0159	0.0351	0.4962
12	2-o-Methylcytidine	0.0001	0.0006	1.0256
13	2-palmitoyl-sn-glycero-3-phosphocholine	0.0001	0.0006	1.2432
14	3-(beta-D-ribofuranosyl)uric acid	0.8118	0.8436	0.1672
15	3,5-dihydroxydecanoic acid	0.0001	0.0006	1.1744
16	3-Amino-3-(4-hydroxyphenyl)propanoic acid	0.0081	0.0209	0.6279
17	3-Indoleacrylic acid	0.0001	0.0006	1.0474
18	4-Aminobutyric acid betaine	0.0002	0.0010	0.6299
19	4-Amino-3-hydroxybutanoic acid	0.6646	0.7338	0.0733
20	5-Aminolevulinic Acid	0.6521	0.7276	0.1653
21	5-Aminovaleric acid	0.0001	0.0006	-1.1943
22	5-Methyl-2'-O-methylcytidine	0.7442	0.7990	-0.0804
23	alpha-D-Glucose	0.0241	0.0511	0.4686
24	Aminocaproic acid	0.0001	0.0006	1.1607

No.	Metabolite	*p*-value	*q*-value	Hedges' *g*
25	Anhydrovitamin A	0.2276	0.3260	0.2755
26	Arachidic acid	0.2182	0.3168	0.2600
27	Benzoic Acid	0.0003	0.0013	0.6979
28	beta-Hydroxymyristic acid	0.0072	0.0191	-0.6533
29	Betaine	0.0032	0.0097	0.6942
30	Bilirubin	0.1062	0.1655	0.3799
31	Caffeine	0.4161	0.5251	0.3477
32	Cortisol	0.2625	0.3614	0.4420
33	Creatine	0.0755	0.1270	0.4929
34	Creatinine	0.0004	0.0016	0.8353
35	Dehydroandrosterone	0.0098	0.0242	-0.5713
36	Deoxyribose 5-phosphate	0.0001	0.0006	0.9180
37	Elaidic Acid	0.0241	0.0511	-0.4517
38	Ergothioneine	0.0480	0.0893	0.2317
39	Estriol	0.0333	0.0654	0.5374
40	Ethyldodecanoate	0.3906	0.4988	0.2199
41	FA 18:1+1O	0.0004	0.0016	0.8914
42	Glyceryl linolenate	0.4375	0.5456	0.1918
43	Glycochenodeoxycholic Acid	0.2372	0.3308	-0.3059
44	Glycocholic Acid	0.8882	0.9053	-0.0756
45	Glycyl-tyrosyl-alanine	0.1314	0.2019	-0.2722
46	Heptadecanoic Acid	0.0001	0.0006	0.8931
47	Heptanoylcarnitine	0.0910	0.1484	0.5447
48	Hypoxanthine	0.0389	0.0750	0.5683
49	Inosine	0.7734	0.8117	0.3994
50	Inosine Diphosphate	0.0303	0.0622	0.5098
51	L-Acetylcarnitine	0.0007	0.0027	0.5515
52	L-Arginine	0.0640	0.1150	0.5116
53	Lauric acid	0.4849	0.5900	-0.1693
54	L-carnitine	0.6036	0.7109	0.1849
55	Leucylproline	0.0032	0.0097	0.5391
56	L-Hexanoylcarnitine	0.0003	0.0013	0.9042
57	Linolenic acid	0.0092	0.0232	0.6271
58	Linoleoylcarnitine	0.0072	0.0191	0.3128

No.	Metabolite	*p*-value	*q*-value	Hedges' *g*
59	L-Phenylalanine	0.0001	0.0006	0.9313
60	L-Proline	0.0001	0.0006	0.9954
61	L-Pyroglutamic Acid	0.0001	0.0006	0.6404
62	LysoPC(0:0/18:0)	0.0001	0.0006	1.2005
63	LysoPC(16:0/0:0)	0.0001	0.0006	1.0688
64	LysoPC(16:1)	0.6294	0.7174	0.1138
65	LysoPC(18:0/0:0)	0.0121	0.0279	0.5528
66	LysoPC(18:1/0:0)	0.0035	0.0103	0.7217
67	LysoPC(18:2/0:0)	0.0002	0.0010	0.7292
68	LysoPC(18:3)	0.1062	0.1655	0.1774
69	LysoPC(20:1/0:0)	0.3430	0.4489	0.1983
70	LysoPE(0:0/18:3)	0.0048	0.0138	-0.6371
71	LysoPE(0:0/20:2(11Z,14Z))	0.7462	0.7990	0.1457
72	LysoPE(16:0/0:0)	0.0735	0.1257	0.4440
73	LysoPE(18:2/0:0)	0.0011	0.0040	0.7262
74	MGMG 18:2	0.0001	0.0006	0.8510
75	Myristoyl Ethanolamide	0.2678	0.3639	0.2689
76	Myristoyl-carnitine	0.0305	0.0622	-0.3033
77	N-Acetylglutamine	0.0446	0.0844	0.4875
78	Nicotinate D-ribonucleotide	0.0001	0.0006	0.8202
79	N-methyl-4-pyridone-3-carboxamide	0.9465	0.9555	-0.1598
80	N-palmitoleoyl-D-erythro-sphingosine	0.0638	0.1150	-0.3426
81	Octanoylcarnitine chloride	0.0984	0.1580	0.3771
82	O-Decanoyl-L-carnitine	0.0658	0.1162	0.4425
83	Oleoyl-L-carnitine	0.7718	0.8117	0.1514
84	Palmitoylcarnitine	0.0057	0.0159	0.2556
85	PC(15:0/0:0)	0.0007	0.0027	0.7615
86	Phenylacetylglutamine	0.3121	0.4188	0.1529
87	Phenylbutyrylglutamine	0.9854	0.9854	0.1394
88	Phosphoric acid	0.0313	0.0626	0.6336
89	phytosphingosine-1-P	0.5741	0.6838	0.1958
90	Pipecolic Acid	0.4898	0.5900	0.1755
91	Propionylcarnitine	0.0003	0.0013	-0.6968
92	PS(17:0/20:4)	0.0016	0.0057	0.7436

No.	Metabolite	*p*-value	*q*-value	Hedges' *g*
93	Pyroglutamylisoleucine	0.2309	0.3263	-0.2965
94	Serotonin	0.0125	0.0282	0.2056
95	Sphinganine	0.0115	0.0271	0.6584
96	Stearoyl Carnitine	0.0002	0.0010	0.2864
97	Thymidine	0.0735	0.1257	-0.3045
98	Trimethylamine oxide	0.3686	0.4765	-0.3648
99	Urea	0.0021	0.0070	0.6937
100	Uric acid	0.0001	0.0006	1.0858
101	Ursocholic acid	0.1916	0.2861	-0.3807

Table S9. Summary of statistical testing results for UPLC-QTOF-MS (negative mode) plasma metabolites between the HC and IBS groups. The table presents raw *p*-values (Welch's t-test or Mann–Whitney U test), FDR-adjusted *q*-values, and effect sizes (Hedges' *g*)

No.	Metabolite	*p*-value	*q*-value	Hedges' *g*
1	1-Heptadecanoyl-sn-glycero-3-phosphocholine	0.4595	0.6211	0.1151
2	3-(Sulfooxy)benzenepropanoic acid	0.7239	0.7818	-0.2317
3	Acarbose	0.4167	0.6211	-0.1967
4	alpha-D-Glucose	0.0026	0.0140	-0.4489
5	Bilirubin	0.4976	0.6211	0.1538
6	Cilastatin	0.4898	0.6211	-0.2385
7	Citric acid	0.0155	0.0465	0.2288
8	Cortisol	0.5291	0.6211	0.3194
9	Cyclamate	0.0001	0.0014	0.7871
10	Docosahexaenoic acid	0.0084	0.0284	-0.5323
11	Glycochenodeoxycholic Acid	0.0959	0.2336	-0.4216
12	Glycocholic Acid	0.8666	0.8666	-0.1719
13	LysoPC(16:1)	0.0026	0.0140	0.7345
14	LysoPC(18:0/0:0)	0.0039	0.0176	0.6417
15	LysoPC(18:1/0:0)	0.1602	0.3234	0.3944
16	LysoPC(18:2/0:0)	0.0007	0.0063	0.7589
17	LysoPC(18:3)	0.6381	0.7179	0.0912
18	LysoPE(0:0/18:3)	0.5140	0.6211	-0.1727
19	LysoPE(16:0/0:0)	0.1038	0.2336	-0.6620
20	LysoPE(18:1/0:0)	0.8403	0.8666	0.0698
21	PC(15:0/0:0)	0.0260	0.0702	0.3904
22	P-Cresol sulfate	0.4822	0.6211	-0.0543
23	Penicillin G	0.1677	0.3234	0.3047
24	Phenol sulfate	0.4161	0.6211	0.2673
25	Urate	0.0001	0.0014	1.0554
26	Verapamil	0.4557	0.6211	-0.5114
27	Zardaverine	0.0069	0.0266	0.6615
